# Identifying psychological distress data available in nationally representative surveys: A scoping review and case study of Australian surveys

**DOI:** 10.1007/s00127-025-02981-6

**Published:** 2025-08-11

**Authors:** D. Varley, A. Henry, J. Halladay, A. Baillie, K. Keyes, T. Slade, C. Chapman, S. O’Dean, R. Visontay, L. Mewton, N. C. Newton, M. Teesson, M. Sunderland

**Affiliations:** 1 The Matilda Centre for Research in Mental Health and Substance Use, The University of Sydney, Sydney, Australia; 2 School of Nursing & Peter Boris Centre for Addictions Research, McMaster University/St. Joseph’s Healthcare Hamilton, ON, Canada; 3 Faculty of Medicine and Health, University of Sydney, Sydney, Australia; 4 Mailman School of Public Health, Columbia University, New York, USA

**Keywords:** Mental health, Epidemiology, Psychological distress, Depression, Systematic reviews

## Abstract

**Purpose:**

Mental health data are crucial for understanding trends in psychological distress. This scoping review aimed to identify and describe surveys of representative samples of the Australian household population that measured psychological distress, and to provide a case study illustrating how datasets can be systematically summarized to assist researchers to more easily identify available datasets.

**Methods:**

We systematically searched PubMed and data archives for surveys state or nationally representative of the Australian household population that assessed psychological distress.

**Results:**

We provide a searchable metadata database characterizing 283 identified datasets from 41 studies (25 cross-sectional, 16 longitudinal) conducted between 1989 and 2023. Thirty-nine psychological distress instruments were used, with the Kessler Psychological Distress scale (K10) [1] most common (*n* = 114 datasets). Surveys also frequently measured demographics, physical health, and socioeconomic information. Stratified random sampling of geographic areas was the most common sampling frame, and adults the most frequently sampled group. There was notably less representation of important subgroups of the population, including youth, Aboriginal and Torres Strait Islander people, and people with disabilities, despite evidence of high distress prevalence in these groups.

**Conclusions:**

This review provides valuable metadata summarizing available psychological distress datasets, including information on sampling designs, instrumentation, and covariates. This metadata is available to other researchers, enabling efficient identification of relevant datasets, promoting data sharing, and supporting future data integration. This method for systematically compiling metadata can be replicated for data related to other topics important to public health to facilitate greater data utilization.

**Supplementary information:**

The online version contains supplementary material available at 10.1007/s00127-025-02981-6.

## Introduction

Poor mental health negatively affects quality of life [2]. In Australia, mental and substance use disorders are responsible for approximately 15% of total burden of disease, making these disorders the second most burdensome disease group [3]. However, this burden extends further, with many undiagnosed individuals or those suffering from symptoms that do not meet the threshold for diagnosis of a mental disorder still negatively affected by their symptoms [4, 5]. Prior evidence demonstrates that high psychological distress, associated with symptoms of anxiety and depression [6, 7], can significantly impair daily functioning, social and emotional wellbeing, and, if untreated, can worsen and create greater negative impact on individuals, families and health care systems [8].

Consequently, measures of psychological distress that effectively capture snapshots of population mental health are included in many epidemiological surveillance studies, national surveys, and longitudinal cohort studies in Australia and elsewhere, even when mental health is not the primary focus of these surveys [9]. Such data offer potential to examine trends in psychological distress. The wealth of available data also provides opportunities to cross-validate findings across datasets. For example, the National Drug Strategy Household Surveys [10] and the Household, Income and Labour Dynamics Survey [11] data both show psychological distress increasing in recent birth cohorts [12, 13].

However, these data are not utilized as well as they could be, and data could be consolidated to more comprehensively and accurately capture trends and identify population-level correlates of psychological distress. Currently, datasets are typically analyzed in isolation. This can provide conflicting information about trends in psychological distress over time. For example, some analyses of repeated cross-sectional survey data (e.g., the National Health Survey/NHS between 2001 and 2014) suggest the prevalence of very high psychological distress has remained stable over time while other analyses of the same data (from 2001 to 2018) and analyses of longitudinal data from the Household Income and Labor Dynamics in Australia Survey (between 2007 and 2017) suggest an increasing trend in the prevalence of very high psychological distress [14, 15]. Critically, very high psychological distress was defined the same way in these surveys, but different conclusions were drawn. Individual surveys are also limited in their ability to capture levels of psychological distress experienced by subpopulations or minority groups with sufficient power. The nature of cross-sectional survey designs and the availability of resources to maximize responses to longitudinal surveys also limits the scope for modelling change across time for people of different ages.

One possible solution is to draw data from multiple sources to create larger, harmonized datasets that could provide more accurate insights into correlates of and trends in psychological distress [16, 17]. However, heterogeneity of survey design, including variability in instrumentation, typically prevent users from better utilizing available data. Fortunately, advances in integrative data analysis, individual participant meta-analysis, and data harmonization methods present promising avenues for addressing such heterogeneity [9, 18–20].

For example, to facilitate pooling and analysis of data from multiple sources, item response theory (IRT) approaches can be used to identify if scale items function differently across different groups of people or samples in different studies (i.e., testing Differential Item Functioning), to place scores for items from different psychological distress measures on the same measurement scale [19, 21–26], and develop crosswalks enabling scores on one measure to be converted to equivalent scores on another measure [9, 27–33]. Other advanced methods, such as non-linear moderated factor analysis (NLMFA), can account for differences in how psychological distress is measured across groups or studies by modeling item functioning as a function of sample characteristics (e.g., age or survey year) [18, 20, 33]. Individual-level data pooled from multiple sources using such approaches can be used in consequent analyses, making valuable reuse of existing data, and providing greater statistical power and capacity to answer questions beyond the scope of individual studies (e.g., examining effects over time by analyzing pooled data from multiple time periods).

To facilitate use of such methods, a scoping review that identifies available data, and summarizes differences in instrumentation, survey design, and availability of covariates is required [34, 35]. However, we are unaware of any review or metadata resource summarizing Australian psychological distress data. Elsewhere, researchers [36] conducted a scoping review categorizing types of mental health data available in Latin American and Caribbean countries. In Australia, other researchers [37] summarized the content surveyed in 13 Australian geo-referenced population health surveys, but they did not focus on or identify all psychological distress data available. Indeed, there is currently no searchable database of metadata summarizing representative psychological distress datasets. Such a resource could facilitate data harmonization, help researchers efficiently identify data aligned with their research needs (e.g., datasets that include covariates of interest), and provide a model illustrating how data can be systematically summarized to increase its identifiability and use. This model can be applied by others to data related to other topics and regions to increase broader data utilization.

Consequently, we conducted a scoping review of psychological distress data from representative Australian surveys. This review aims to enable harmonization, encourage greater use of psychological distress data, and provide a model for creation of metadata resources that can increase identifiability and use of data related to other regions and topics. Our objectives were to: (1) identify surveys representative of the general Australian household population that contained at least one measure of psychological distress and/or a measure of symptoms of depression, anxiety and/or stress (as these are considered subcomponents of psychological distress) [6, 7]; and (2) summarize key characteristics of these surveys, including instruments used to measure psychological distress, survey/study designs, sampling designs, and covariates/other domains assessed in these surveys.

## Method

We followed the Preferred Reporting Items for Systematic Reviews and Meta-Analyses extension for Scoping Reviews (PRISMA-ScR) guidelines for reporting findings [38]. The protocol was planned but not pre-registered.

### Eligibility criteria

Surveys were eligible for inclusion if they met the following criteria:

1. The surveys were representative at an Australian national or state level, even if the survey only collected data from a major demographic subgroup of the population (e.g., women, older adults).
2. The survey included a self-reported measure of psychological distress (e.g., the Kessler Psychological Distress scale; K10) [1].

Surveys that collected data from a population subgroup were considered eligible if data were considered likely to be broadly characteristic of the general Australian population context or if the subgroup represents a large portion of the population. For example, surveys using representative samples of women or older adults were included. However, surveys that sampled narrowly defined subgroups (e.g., clinical in-patients, people with a specific disease) which may not be broadly characteristic of the general Australian population context were excluded.

We determined whether surveys were representative using information in survey documentation. To be considered representative, surveys needed to have taken steps to ensure sample characteristics were similar to population estimates (e.g., census data estimates) through sampling methods that facilitate proportional representation across key demographic characteristics (e.g., stratification, quota sampling, random sampling) and/or use of weighting procedures. 

### Information sources and search strategy

We conducted a systematic search of PubMed on February 13th, 2024, to identify potentially eligible survey datasets. We used search terms related to psychological distress and mental health and search terms designed to identify cross-sectional and longitudinal Australian surveys (see Online Resource 1 for full search terms). MS developed and adapted the search terms based on a systematic review summarizing mental health data available in Latin-America [36]. We also contacted known data custodians and reviewed known data archives (Australian Data Archive, the Australian Bureau of Statistics MicroData Download archive, and the LifeCourse data archive).

### **Selection of sources of evidence**

**Fig. 1:**
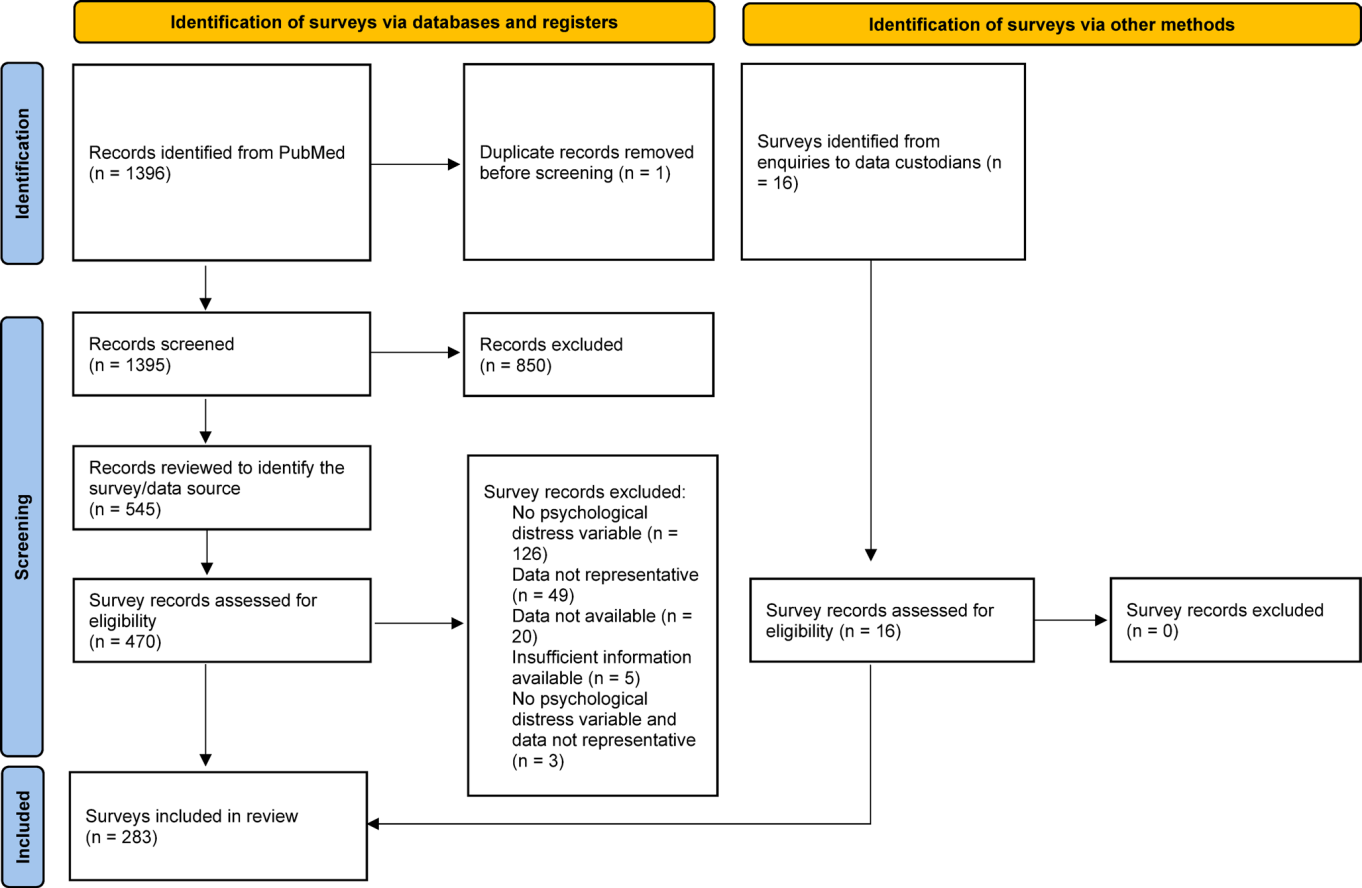
PRISMA 2020 flow diagram illustrating screening and selection of records and surveys

A total of 1396 entries were identified from PubMed and uploaded into Rayyan systematic review software [39]. We also identified 16 surveys via enquiries to data custodians. One duplicate was removed. MS screened titles and abstracts, with 850 entries excluded and 545 progressing to full text review, where each entry was examined to identify potentially eligible datasets. From this, 470 identified data sources (i.e., surveys) were extracted into a spreadsheet. MS reviewed documentation for each survey dataset (i.e., data dictionaries, questionnaires, reports) to determine eligibility. DV also independently screened a random sample of 20% of these records. Overall, 283 eligible surveys were identified (see Fig. 1). Interrater agreement indices for each eligibility criterion are in Table 1. For any disagreements, MS and DV discussed and reviewed information related to that survey again to ensure an accurate final decision.

**Table 1:** Inter-rater agreement for decisions for each eligibility criterion and for data extraction of each data item

Variable	Percentage agreement (%)		Kappa (k)
Agreement for eligibility criteria decisions			
Representativeness		100.0%	1.00^**‡**^
Psychological distress variable available		90.8%	0.73^†^
Inclusion/exclusion decision		91.3%	0.82^†^
Agreement for extracted data items			
Year of data collection		100.0%	1.00^**‡**^
Study design		100.0%	1.00^**‡**^
Region sampled		100.0%	1.00^**‡**^
Sampling design		100.0%	1.00^**‡**^
Population		100.0%	1.00^**‡**^
Minimum age		100.0%	1.00^**‡**^
Maximum age		89.30%	0.88^**‡**^
Psychological distress instrument		89.70%	0.88^**‡**^
Other surveyed domains		93.30%	0.93^**‡**^

^†^Indicates substantial agreement according to rules of thumb [40] for Cohen’s Kappa (k) [41]. ^**‡**^Indicates almost perfect or perfect agreement according to rules of thumb for Cohen’s Kappa.

### **Data items**

Data were extracted from survey documentation (e.g., data dictionaries, questionnaires, reports) by DV and AH using a data extraction form (see Online Resource 2) created by MS and refined by DV after piloting data extraction for 25% of eligible surveys. DV and AH piloted data extraction for a further 5% of surveys together to ensure consistency. DV and AH extracted all other data independently and double-coded a random sample of 10% of surveys to evaluate interrater agreement for extracted data items (see Table 1). Cohen’s Kappa coefficients indicated almost perfect or perfect agreement for all double-coded data items. Additional meta-data extracted by DV included links to manuals/user guides/reports, whether the dataset is accessible, and links to available datasets.

To summarize domains other than psychological distress measured in each survey, DV compiled a list of every variable measured in a sample of 25% of all included surveys. DV and MS reviewed this information and used inductive coding to derive codes/categories to summarize the domains assessed. DV and AH used this coding frame to record which domains were measured in each survey. A detailed description of the coding frame and variables included in each category is in Online Resource 1.

Any doubts during data extraction were resolved in team meetings. If published information was insufficient to complete data extraction, DV contacted the relevant data custodian or corresponding authors twice to request further information. Surveys without sufficient published information to extract certain data items were marked ‘More information required’ if no response was received by November 1, 2024.

### **Quality assessment**

Quality assessments are optional for scoping reviews [38] and were not feasible for this review. Quality assessments typically require evaluation of theoretical grounding, appropriateness of methods to address research questions, other study-specific details, and the reporting of details in articles. This was not possible as we aimed to review datasets rather than studies. Additionally, most datasets were from epidemiological/public health surveys which have numerous aims and often don’t summarize study details in articles to facilitate evaluation of characteristics like theoretical grounding or the quality of reporting. However, we encourage researchers interested in using the datasets to consider the quality of methods used with reference to their requirements.

### Synthesis of results

Using the extracted data, DV used Microsoft Excel and R (Version 4.4.0) [42] to create tables, narrative summaries (i.e., written descriptions of results) and data visualizations of key information including region sampled, representativeness, populations sampled, sampling designs, data availability, psychological distress instrumentation, and other domains assessed. DV also used R to develop an interactive platform that allows users to search the metadata compiled for this review (https://deannavarley.shinyapps.io/the_australian_psychological_distress_data_search_tool/). This tool enables users to filter, browse, and view information summarizing the features of the datasets identified in this review.

## Results

### Sources of evidence

We identified 283 datasets, with 125 datasets from repeated or one-off surveys from 25 cross-sectional studies and 158 datasets from 27 cohorts from 16 longitudinal studies.

### **Characteristics of surveys**

Key characteristics of the survey datasets are summarized below and in Table 2. Additional summary tables are in Online Resource 1, the full metadata database we compiled is in Online Resource 2, and an interactive online platform that allows users to search the metadata database to facilitate identification of datasets meeting researchers’ criteria is available here: https://deannavarley.shinyapps.io/the_australian_psychological_distress_data_search_tool/.

**Fig. 2:**
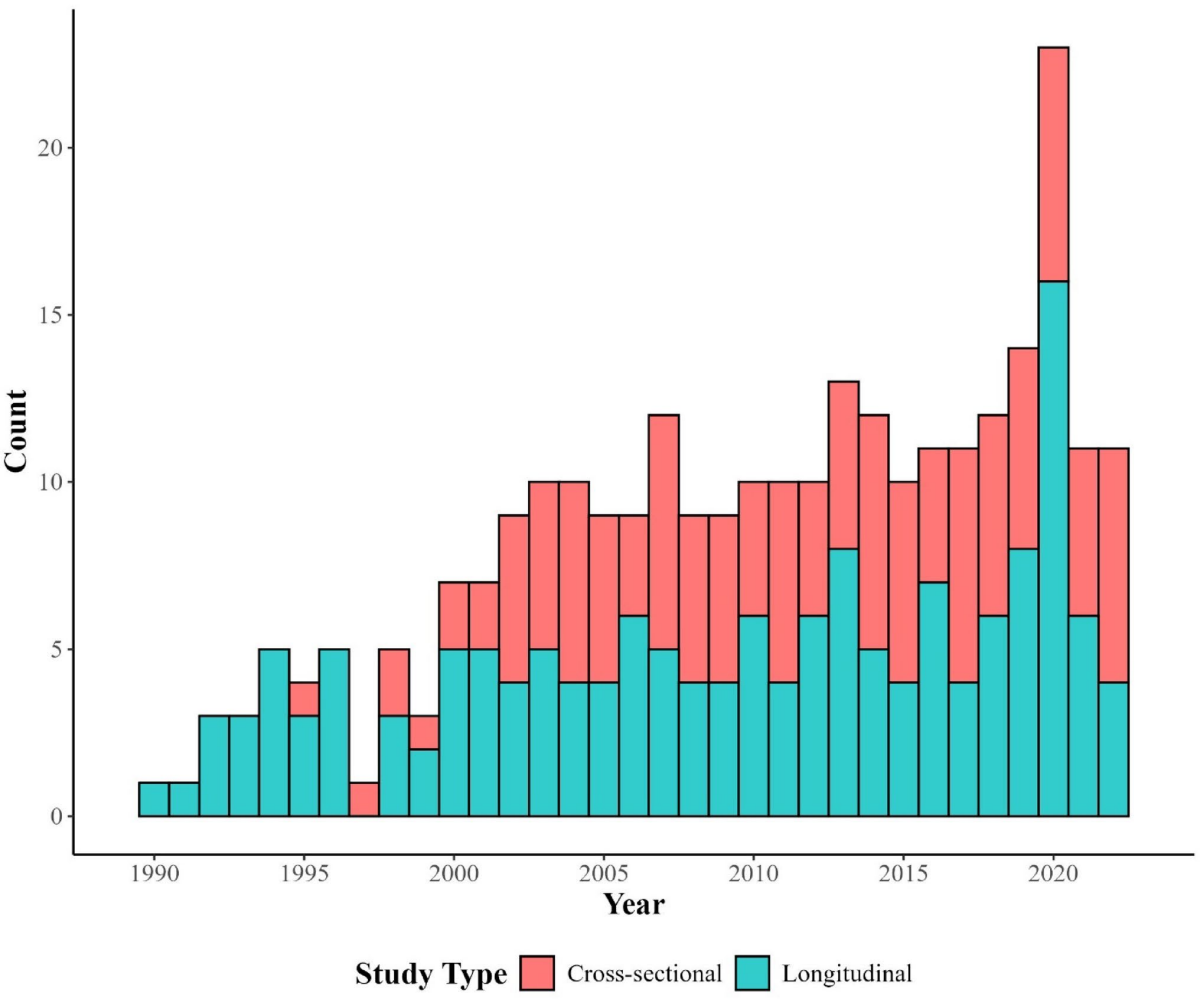
Histogram of year of data collection for all cross-sectional surveys and all waves of all longitudinal surveys

We identified data collected from 1989 to 2023, with cross-sectional data from 1995 to 2023 and longitudinal data from 1989 to 2023. The number of surveys peaked in 2020, with 24 datasets available (see Fig. 2). The number of cross-sectional surveys was equally highest in 2007, 2014, 2017, 2020 and 2022, with 7 datasets from each year. The number of longitudinal surveys was highest in 2020, with 17 datasets available.

Overall, 147 (51.94%) survey datasets were nationally representative, while 136 (48.06%) were representative on a state level. Other than national surveys, there were no surveys with data drawn from Queensland or Tasmania, and only one with data from the Northern Territory.

To select representative samples, 197 (69.61%) datasets used stratification (sometimes combined with weighting), while the remaining 86 (30.39%) used other methods like weighting, raking, or quota sampling. The most common sampling design was stratified random sampling of statistical geographic areas (38.87%), followed by stratified random sampling via other methods (e.g., random digit dialing; 16.61%). Adults with a fixed address (excluding those without fixed addresses, in residential care, or incarcerated) were the most frequently sampled population in both cross-sectional and longitudinal surveys, appearing in 53.36% of surveys.

Survey documentation limitations prevented us from precisely calculating the age range for which self-report psychological distress data are available, as some surveys don’t specify participants’ maximum age or the age when child participants, rather than proxies (e.g., parents), provided data. However, estimations suggest that self-report cross-sectional data is available for participants from 11 to at least 80 years old, and longitudinal data is available for participants 11 to at least 100 years old.

**Table 2:** Survey characteristics

		Cross-Sectional		Longitudinal		All Surveys	
		*N*	%	*N*	%	*N*	%
Region							
	National	42	33.60	105	66.46	147	51.94
	State (Victoria)	21	16.80	27	17.09	48	16.96
	State (New South Wales)	32	25.60	3	1.90	35	12.37
	State (Western Australia)	22	17.60	0	0.00	22	7.77
	State (Australian Capital Territory)	0	0.00	15	9.49	15	5.30
	State (South Australia)	6	4.80	8	5.06	14	4.95
	Multi-state (New South Wales and Victoria)	1	0.80	0	0.00	1	0.35
	Multi-state (Western Australia, Northern Territory, South Australia)	1	0.80	0	0.00	1	0.35
Population represented^†^							
	Adults (18 + years)	98	78.40	53	33.54	151	53.36
	Adolescents (13–17 years)	46	36.80	38	24.05	84	29.68
	Children (≤ 12 years)	20	16.00	26	16.46	46	16.25
	Older adults (65 + years)	10	8.00	34	21.52	44	15.55
	Female adults and female older adults (18 + years)	0	0.00	32	20.25	32	11.31
	Male children (≤ 12 years)	0	0.00	4	2.53	4	1.41
	Male adults (18 + years)	0	0.00	4	2.53	4	1.41
	Aboriginal or Torres Strait Islander adolescents (13–17 years)	1	0.80	2	1.27	3	1.06
	Aboriginal or Torres Strait Islander adults (18 + years)	5	4.00	2	1.27	7	2.47
	Aboriginal or Torres Strait Islander children (≤ 12 years)	1	0.80	5	3.16	6	2.12
	People with disability	6	4.80	0	0.00	6	2.12
	Carers of people with disability	6	4.80	0	0.00	6	2.12
Sampling design							
	Stratified area sampling	45	36.00	65	41.14	110	38.87
	Stratified random sampling	29	23.20	18	11.39	47	16.61
	Stratified cluster sampling	7	5.60	33	20.89	40	14.13
	Non-stratified random sampling	8	6.40	27	17.09	35	12.37
	Non-stratified area sampling	35	28.00	0	0.00	35	12.37
	Quota sampling	0	0.00	8	5.06	8	2.83
	Non-stratified purposive sampling	0	0.00	5	3.16	5	1.77
	Non-stratified cluster sampling	0	0.00	2	1.27	2	0.71
	Census sampling	1	0.80	0	0.00	1	0.35
Dataset availability							
	Available	117	93.60	154	97.47	271	95.76
	Under embargo	2	1.60	2	1.27	4	1.41
	More information required	6	4.80	2	1.27	8	2.83
Data available only via secure access environment							
	More information required	7	5.6	38	24.05	45	15.90
	No	80	64.0	117	74.05	197	69.61
	Yes	38	30.40	3	1.90	41	14.49

^†^Percentages for ‘Population Represented’ reflect the proportion of surveys that sampled each population. A sum of the percentage values of greater than 100% is possible as several datasets sampled more than one population. Surveys were counted as having sampled a population subgroup if they self-described collecting a representative sample of that group (or this could be reasonably inferred) and they measured psychological distress in that group. More detailed population information is available in the Online Resources

Overall, 95.76% of datasets are available via applications to custodians or for download by approved users of systems like the Australian Bureau of Statistics’ MicrodataDownload service [43]. The remaining datasets were either under embargo or availability was unclear. Further, at least 14.49% of datasets are only accessible via secure access environments (i.e., protected computing environments that ensure confidentiality and data integrity by restricting access and enforcing security measures).

### **Psychological distress instruments**

Across datasets, psychological distress was measured 460 times, with 28 validated instruments used for 412 measurements and 11 other instruments used for 48 measurements. The other instruments identified included seven unique single-items, and four unique sets of two or more items. Survey documentation did not report their origin.

**Fig. 3:**
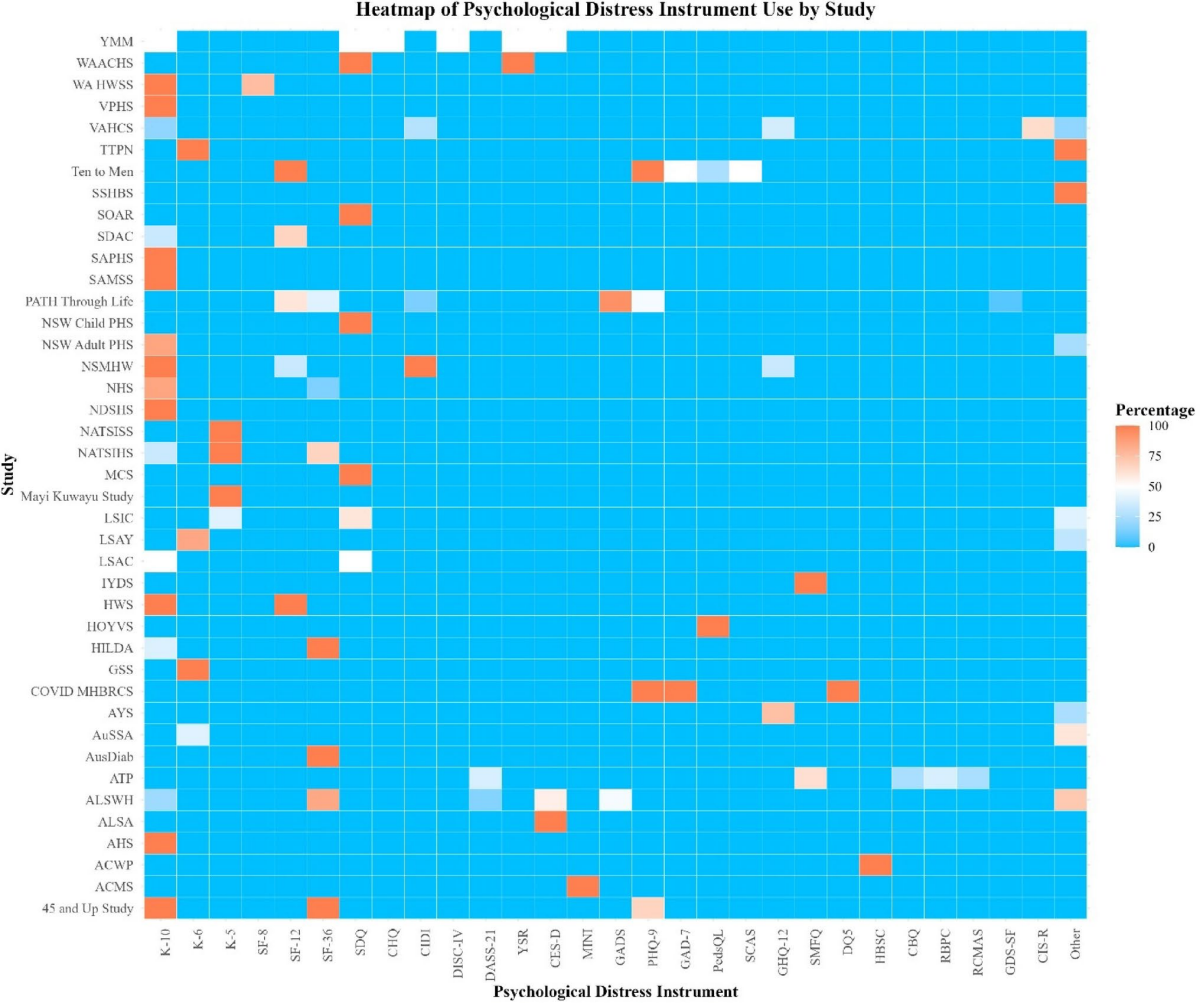
Heatmap of psychological distress instrument use in each study. *Note*: Percentages indicate the proportion of the waves/repeats of each study in which a given instrument was used. For definitions of acronyms and abbreviations used for the names of studies and psychological distress instruments see Online Resource 1. Versions of the same scale that differ in length (e.g., K10, K6, K5) are shown separately. However, studies using longer forms may be considered to implicitly include shorter forms because short form items are derived from longer versions (though item content/wording sometimes vary)

The K10 [1] was the most frequently used scale, appearing in 114 (40.28%) surveys and accounting for 24.78% of all measurements of psychological distress. If including short forms of the K10 (K6, K5), this increases further, with use in 139 (49.11%) surveys and 30.22% of measurements. This was followed by the Medical Outcomes Study 36-Item Short-Form Health Survey (SF-36) [44], included in 63 surveys (22.26%) and comprising 13.70% of measurements. Frequency of instrument usage by study is visualized in Fig. 3 (note: several surveys included more than one measure of psychological distress). An interactive version of Fig. 3 and detailed summary table are available in Online Resource 1. Characteristics of the identified instruments are summarized in Online Resource 1.

### **Other surveyed domains**

We identified 13 domains surveyed in addition to psychological distress. The most frequently surveyed additional domain was demographic characteristics (100% of datasets), followed by physical health (99.29%), socioeconomic information (95.05%), other mental health-related variables (e.g., wellbeing; 94.35%), alcohol and/or drug use (78.80%), social wellbeing (75.62%), biometric measurements (e.g., BMI, blood pressure; 66.43%), major or adverse life events (61.13%), disability-related variables (59.01%), caring responsibilities (e.g., time caring for children; 52.65%), social attitudes (e.g., political views; 46.64%), personality (43.11%), and technology use (e.g., screen time; 30.74%).

**Fig. 4:**
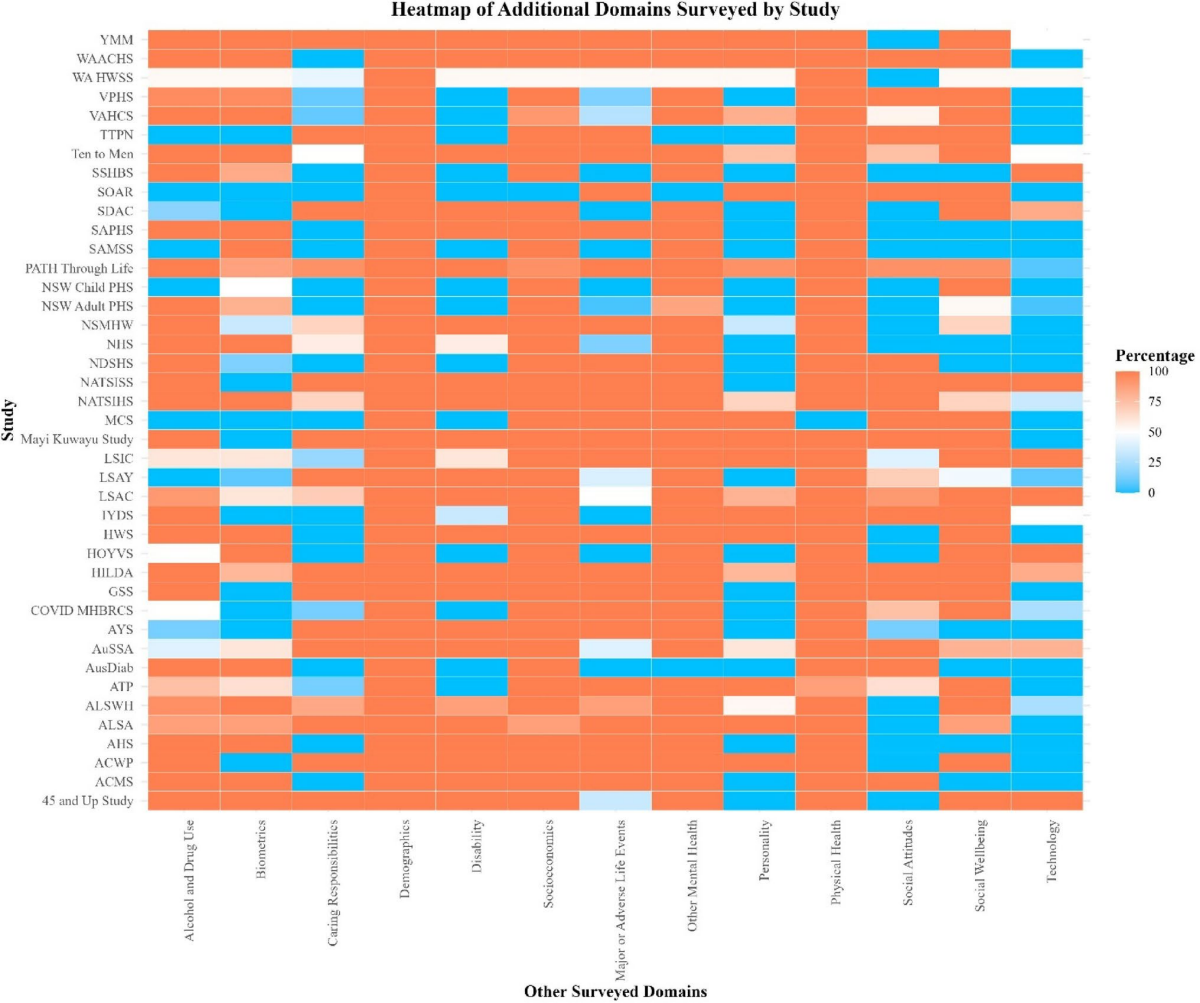
Heatmap of other surveyed domains in each study. *Note*: Percentages indicate the proportion of the waves/repeats of each study in which a given domain was surveyed

The proportions of the waves/repeats of each study in which a given domain was surveyed are visualized in Fig. 4. See Online Resource 1 for an interactive version of this figure and a table summarizing coverage of each domain in cross-sectional versus longitudinal datasets.

## **Discussion**

We aimed to improve utilization of psychological distress data, facilitate future data harmonization, and provide a model illustrating how data can be systematically summarized to facilitate greater data use. We identified and summarized the characteristics of psychological distress data from 283 representative Australian survey datasets from 41 studies.

Most surveys used stratification to select representative samples, with stratified random area sampling the most common sampling design. Approximately half the datasets were nationally representative, and half representative of one or more states/territories. However, we are not aware of any established method for evaluating and comparing representativeness across surveys, and it should be noted that samples self-described by researchers as representative can vary in their similarity to the population they are intended to represent. Surveys using more stringent sampling methods arguably yield more representative samples, but those wishing to use datasets identified in this review should remember that true representativeness can vary and assess whether the sampling and weighting methods used meet their requirements.

Across the datasets, psychological distress was measured 460 times using 39 instruments. Of these, 11 were unvalidated. Occasional use of unvalidated instruments is a limitation of some surveys, potentially compromising the reliability and validity of data. The remaining 29 instruments were validated, with the K10 the most prevalent, highlighting a preference for the K10 across surveys. However, there is notable heterogeneity in psychological distress instrumentation overall. This is an understandable consequence of there being few comparative evaluations of psychological distress measures [9], and differing aims and resources across surveys. However, heterogenous instrumentation can prevent integration and comparison across datasets to answer large-scale questions about psychological distress in the population [15, 45]. Fortunately, data harmonization methods could address these issues.

Beyond psychological distress, we identified 13 other surveyed domains, the most common of which were demographic characteristics, physical health, and socioeconomic information. These findings correspond with a review of content in Australian population health surveys identifying that Australian population health surveys data generally collect data related to demographic characteristics, physical health risk factors and self-rated physical health [37]. We also identified that domains like personality and technology use were measured less often across surveys. This coverage gap persists despite research connecting personality and problematic technology use with psychological distress and poorer mental health [46–49]. Surveys would benefit from more frequently inclusion of measures of these domains. Such data could provide valuable insights for understanding, addressing, and planning for these domains as risk factors for psychological distress. While it can be unfeasible to measure all relevant covariates of psychological distress, more surveys could measure these domains using short scales. Short scales still offer significant utility, especially when harmonized with longer, more comprehensive assessments using data harmonization methods.

There was also less data available for some regions and subpopulation groups. The only data available for the states/territories of Queensland, Tasmania and the Northern Territory were drawn from national or multi-state surveys. Additionally, most surveys do not collect data from very remote areas. Consequently, there is less psychological distress data for these regions relative to others. This is noteworthy as Queensland, Tasmania and the Northern Territory represent almost a quarter (23.57%) of the Australian population [50]. Further, while only a small portion of the population live in very remote areas, approximately 23.00% of the Northern Territory population live in very remote areas. Consequently, available data may provide less accurate estimates of psychological distress for the Northern Territory. This issue may also be greater for Aboriginal and Torres Strait Islander people, who make up 89.60% of people living in very remote areas of the Northern Territory [51].

Further, very few surveys recruited representative samples of important subgroups of the Australian population including Aboriginal or Torres Strait Islander people and people with disabilities, and no surveys recruited representative samples of refugees, people from culturally and linguistically diverse backgrounds, or people without a fixed address. While these individuals are sometimes included in broader population samples, methodological and resource limitations mean they are often under-sampled, and many surveys specifically exclude these groups. Consequently, these groups are likely underrepresented in available data. This observation aligns with longstanding concerns about underrepresentation of minority or socially disadvantaged groups in public health and mental health research [52, 53]. Underrepresentation reduces the accuracy of estimates of psychological distress in these groups and the Australian population in general, especially as these groups are not always accounted for in weighting procedures that aim to adjust for sampling bias. Indeed, some surveys such as the National Study of Mental Health and Wellbeing (NSMHWB) acknowledge that exclusion of groups such as people experiencing homelessness may lead to underestimation of the prevalence of psychological distress and mental disorders in Australia [54]. Future studies should aim to address these gaps.

More broadly, representativeness in mental health survey data due to selection and response biases is a long-standing concern for researchers [55–57] that may contribute to varying prevalence estimates across data sources [15, 58]. While methodological differences limit comparison, surveys focused on mental health sometimes report higher prevalence of mental disorders than general surveys (e.g., the NSMHWB versus NHS) [59–61]. Distress and lived experience of mental health difficulties have also been associated with greater willingness to participate in mental health research [62–65], and self-selected participants report higher distress compared to researcher-selected participants [66]. Consequently, including psychological distress and other mental health measures in general-purpose surveys could support identification of cross-survey differences that may indicate bias, calculation of prevalence estimates in samples potentially less affected by biases, and evidence triangulation [67, 68]. However, representativeness is also a concern for general surveys, with high distress and mental health diagnoses associated with non-response and attrition [69–81]. Together, these challenges underscore the importance of approaches that minimize or adjust for selection and response biases (e.g., sampling strategies, inverse probability weighting). Data users should keep these considerations in mind when selecting and using survey data.

We also found that 14.49% of datasets were restricted to secure access environments/trusted research environments [82, 83]. Researchers may wish to consider the useability of these datasets with reference to their requirements. While secure access environments provide assurance of data security [82, 83], they also impose limitations that can reduce useability and prevent inclusion of datasets in some research projects including projects requiring timeliness, large teams, inter-institutional collaboration, data linkage, or integrated analysis across datasets held by more than one custodian. Additionally, researchers are often required to pay fees to use secure access environments. This may prevent those with funding constraints from using these datasets.

We also note that those interested in data related to Aboriginal and Torres Strait Islander people should be aware of Indigenous Data Sovereignty and Indigenous Data Governance principles. These principles emphasize the right of Aboriginal and Torres Strait Islander people to have ownership and control over data that are about or affect them throughout all stages of the data lifecycle [84]. This helps ensure that data is used in ways that reflect the values, priorities, worldviews, cultures and diversity of Aboriginal and Torres Strait Islander people. Potential data users are advised to consult the relevant frameworks and adhere to all guidelines if interested in this data.

## Strengths and Limitations

One of the key strengths of this review is the detailed cataloging of metadata from 283 Australian survey datasets measuring psychological distress. Much of this information is time-consuming to identify, and occasionally requires enquiries to data custodians. Summarizing this information in one place will save researchers time and enable them to more efficiently identify and access relevant data, including datasets with covariates relevant to their research. The review also identifies limitations in survey designs, sampling, and assessment of covariates of psychological distress. Overall, this information is useful for facilitating future research, identifying gaps in current data coverage and highlighting where further work is required to address gaps in sampling and survey content coverage.

Our review also summarizes instruments used to measure psychological distress in Australia, offering a clear view of available and commonly used scales. This highlights where work is needed to confirm the validity of some instruments and can assist with designing future surveys with reference to researcher’s requirements and desires to compare results to previous findings.

Additionally, this review facilitates future data harmonization by identifying and summarizing datasets available for integration. This will make content analyses required for data harmonization easier and allow identification of covariates available for future analyses of trends in psychological distress.

However, some limitations should be considered. First, we searched PubMed but did not search additional research-focused databases (e.g., PsycINFO). Consequently, a few datasets may have been missed. However, our search strategy suited our aim to identify state/nationally representative datasets, which are generally included in population-level public health/epidemiological surveys, rather than academic studies. Additionally, we searched government and institutional websites/known data archives and checked the content and reference lists of survey documentation and publications from included datasets for references to other possibly eligible datasets.

Second, we used broad categories when cataloging what domains beyond psychological distress were measured in surveys. These categories will help researchers identify datasets relevant to their research needs more efficiently. However, researchers will still need to consult survey documentation to confirm the presence of specific variables and the overall suitability of datasets for their requirements.

Third, we didn’t extract metadata related to sample sizes, gender distribution, or missingness due to time and resource constraints, inconsistent reporting, and complexity. Sample size reporting varied across studies, with some reporting sample size before or after diverse reasons for exclusion, and longitudinal studies often only reporting sample size for the first wave, meaning accurate extraction would require direct dataset access or contacting data custodians. Further, information on missingness was often unavailable, inconsistently reported, or reported for individual variables, making standardized extraction and comparison unfeasible. Gender distribution reporting was also inconsistent (including across waves of the same study). Some studies only reported binary gender identities or did not clarify whether they reported biological sex or gender (e.g., sometimes using terms like ‘women’ interchangeably with ‘females’), while others recognized other gender identities or clearly distinguished between gender and sex. Additionally, definitions of gender varied and were often missing in study documentation and participant questionnaires. These inconsistencies made standardized extraction and comparison across studies unfeasible within the project’s scope and constraints. This prevented us from making inferences about the coverage of Australian psychological distress data related to these characteristics. However, interested readers can consult the documentation of each dataset for further information.

## **Conclusion**

Overall, this scoping review provides a valuable resource for data users, researchers, and those planning future epidemiological/public health surveys. This work not only enhances immediate research capabilities but also highlights gaps in current data and lays a foundation for more effective data integration, ultimately advancing research that will improve our understanding and management of psychological distress in Australia. We encourage other researchers to develop similar metadata resources focused on other topics and region. This would enhance research capabilities for other topics important to public health and allow researchers to more fully leverage available data. 

## Supplementary information

Below is the link to the electronic supplementary material.

Supplementary Material 1

Supplementary Material 2

Supplementary Material 3

Supplementary Material 4

## Data Availability

The data generated by this review are provided in the supplementary materials.
