# Supplementary material for: Identifying psychological distress data available in nationally representative surveys: A scoping review and case study of Australian surveys
Source: Soc Psychiatry Psychiatr Epidemiol. 2025 Aug 11;61(1):1–14. doi: 10.1007/s00127-025-02981-6 (PMC12855228; doi:10.1007/s00127-025-02981-6)
Supplement: Supplementary file 1 — Supplementary Material 1 [file 127_2025_2981_MOESM1_ESM.docx]

**Online Resource 1**

**Table of Contents**

[Search Terms 3](#_Toc202208550)

[Coding Frame of Other Surveyed Domains 4](#_Toc202208551)

[Detailed Breakdown of Other Surveyed Domains 4](#_Toc202208552)

[Demographics 4](#_Toc202208553)

[Physical Health 5](#_Toc202208554)

[Other Mental Health, Wellbeing and Cognitive Function 6](#_Toc202208555)

[Alcohol or Drug/Substance Use 7](#_Toc202208556)

[Social Wellbeing 8](#_Toc202208557)

[Financial and Socioeconomic Information 9](#_Toc202208558)

[Social Attitudes, Beliefs, Perceptions, Political Views 9](#_Toc202208559)

[Disability 11](#_Toc202208560)

[Caring/Caregiving/Household Responsibilities 12](#_Toc202208561)

[Personality 12](#_Toc202208562)

[Physical and Biomedical Measurements 12](#_Toc202208563)

[Technology 12](#_Toc202208564)

[Major Life Events/Adverse Life Experiences/Victimisation 13](#_Toc202208565)

[Definitions of Study Name Acronyms and Abbreviations 14](#_Toc202208566)

[Table S1 14](#_Toc202208567)

[Definitions of Psychological Distress Instrument Acronyms and Abbreviations 15](#_Toc202208568)

[Table S2 15](#_Toc202208569)

[Interactive Figures 17](#_Toc202208570)

[Figure S1 17](#_Toc202208571)

[Figure S2 17](#_Toc202208572)

[Figure S3 17](#_Toc202208573)

[Figure S4 17](#_Toc202208574)

[Figure S5 17](#_Toc202208575)

[Additional Summary Tables 18](#_Toc202208576)

[Table S3 18](#_Toc202208577)

[Table S4 18](#_Toc202208578)

[Table S5 20](#_Toc202208579)

[Table S6 21](#_Toc202208580)

[Table S7 22](#_Toc202208581)

[Table S8 22](#_Toc202208582)

[Table S9 23](#_Toc202208583)

[Table S10 25](#_Toc202208584)

[Table S11 25](#_Toc202208585)

[Characteristics of Psychological Distress Scales 27](#_Toc202208586)

[Table S12 27](#_Toc202208587)

[References for Supplementary Document 46](#_Toc202208588)

# Search Terms

(("mental health"[tiab] OR "psychological health"[tiab] OR "Mental health" [Mesh] OR "Behavioral Symptoms"[Mesh] OR "Mental Disorders"[Mesh] OR "mental disorder*"[tiab] OR "self injur*"[tiab] OR "self mutilat*"[tiab] OR suicid*[tiab] OR parasuicid*[tiab] OR "mood disorder*"[tiab] OR "affective disorder*"[tiab] OR anxiet*[tiab] OR Distress*[tiab] OR "Depressive Disorder"[mesh] OR "Anxiety Disorders"[mesh] OR "Anxiety"[mesh] OR "Psychological Distress"[mesh]) AND ("health survey*"[tiab] OR "national survey*"[tiab] OR “population survey*”[tiab] OR “population”[tiab] OR "mental health survey*"[tiab] OR "Censuses"[Mesh] OR "Cross-Sectional Studies"[Mesh] OR "Health Surveys"[Mesh] OR "Behavioral Risk Factor Surveillance System"[Mesh] OR "Health Status Indicators"[Mesh] OR "Population Surveillance"[Mesh] OR "cohort"[tiab] OR "Longitudinal"[tiab] or “Prospective”[tiab]) ) AND ("Australia"[tiab] OR "Australian"[tiab] OR "New South Wales*"[tiab] OR "Queensland"[tiab] OR "Victoria"[tiab] OR "South Australia*"[tiab] OR "Northern Territory*"[tiab] OR "Western Australia*"[tiab] OR "Tasmania"[tiab]) AND (representative)

# Coding Frame of Other Surveyed Domains

The below is a list of the categories derived from inductive coding of qualitative data related to other domains assessed in the identified surveys. Inductive coding was conducted after extracting detailed qualitative data related to these domains from 25% of the identified surveys. This qualitative data is available in the data extraction form also included in the Supplementary Materials. A detailed list of the constructs/variables included within each category of the coding frame is also provided below.

1. Demographics
2. Physical Health
3. Mental Health, Wellbeing, and Cognitive function
4. Alcohol or Drug/Substance Use
5. Social Wellbeing
6. Financial and Socioeconomic Information
7. Social Attitudes, Beliefs, and Political Views
8. Disability
9. Caring/Caregiving/Household Responsibilities
10. Personality
11. Physical and Biomedical Measurements
12. Technology
13. Major Life Events/Adverse Life Experiences/Victimisation

# Detailed Breakdown of Other Surveyed Domains

## Demographics

- Age
- Sex
- Gender
- Trans/Gender-diverse person status
- Sexuality/sexual orientation
- Education
- Employment
- Marital status
- Languages spoken
- Cultural identification
- Geographic characteristics/location/region
- Household information (number of people in the house, relationships between people in the house etc.)
- Living arrangements
- Mobility (moving house/area)
- COVID-19-related employment changes
- Housing information (home ownership/renting, number of bedrooms in house etc.)
- Family ancestry (where family is from)
- Aboriginal and Torres Strait Islander person status
- Visa Status
- Defence Force service history
- Voluntary work
- Information about the proxy (if a survey was completed on behalf of a child)
- Information about the main carer of the child (if survey respondent was a child/survey was completed on behalf of a child)

## Physical Health

- Health service utilisation
- Physical health
- Health risk behaviours
- Obesity
- Long-term/Chronic health conditions
- Sexually transmitted infections
- Complications of diabetes
- Family health history
- Personal health history
- Health behaviours
- Health risk factors
- Nutrition
- Medications and supplements
- Breastfeeding
- Physical activity
- Self-assessed health status
- Female life stage
- Disordered eating
- COVID-19 vaccine status
- Injuries
- Adult immunisation
- Child immunisation
- Women's health information
- Contraception
- Days off work/study due to illness/injury
- Sleep behaviour
- Daytime sleepiness
- Maternal health status (health of child's mother if the respondent was a child/survey was being completed on behalf of a child)
- Child nutrition, sleep and exercise
- Sun protection
- Diabetes health knowledge
- Health knowledge and attitudes
- Knowledge of drug/alcohol-related information

## Other Mental Health, Wellbeing and Cognitive Function

- Symptoms of psychosis
- Life satisfaction
- Quality of life
- Perceived need for services
- Self-esteem
- Mental health condition/mental health disorder diagnoses
- Emotional wellbeing
- Hypochondriasis/somatisation
- Symptoms of psychosis
- Mental disorder treatment history
- Family mental health history
- Mental health self-management behaviours
- Mental health service utilisation
- COVID-19-related mental health service utilisation
- Disordered eating
- Suicide/self-harm behaviours
- Emotions experienced
- Emotional symptoms
- Conduct problems
- Psychotic-like experiences
- Positive wellbeing
- Mastery
- Connection to nature
- Positive life events of child (e.g., going on a holiday)
- Conduct problems/aggression
- Youth risk behaviours
- Protective behaviours
- Perceptual sensitivity
- Cognitive impairment
- Attention, Inhibitory control, Hyperactivity/Inattention

## Alcohol or Drug/Substance Use

- Tobacco use/Smoking
- Alcohol use
- Cannabis dependence
- E-cigarette usage/vaping
- Other drug/substance use
- Polysubstance use
- Access to drugs/alcohol/tobacco
- Drug/tobacco use in friends/family/household
- Prescription medication use/abuse
- Missed work/study due to drug or alcohol use
- Missed work/study due to illness/injury related to drug/alcohol use
- Drug service utilisation (e.g., drug treatment programs)
- Drug/alcohol use while pregnant/breastfeeding
- Knowledge of drug/alcohol-related information
- Youth risk behaviours

## Social Wellbeing

- Social wellbeing
- Family factual information
- Friends factual information
- School-related factual information
- Community-related factual information
- Family wellbeing
- Friend-related wellbeing
- School-related wellbeing
- Closeness of relationships
- Social networks
- Perceived social support
- Perceived social resources
- Loneliness
- Social engagement
- Sense of belonging
- Community Participation
- Social integration/school-related wellbeing
- Supportive home relationships
- Supportive school relationships
- Supportive community relationships
- Peer relationship problems
- Aggression
- Prosocial behaviours

## Financial and Socioeconomic Information

- Money and material wellbeing/socioeconomic evaluation
- Personal income
- Household income
- Financial stress and resilience
- Service utilisation (social support services)
- Private health insurance status
- Financial status (e.g., perceived quality of financial position)
- Perceived standard of living
- House and contents insurance
- Social and financial impacts of the COVID-19 pandemic
- Value of Home and Household Assets and Liabilities
- Receipt of/persistence of Government Payments
- Food security
- Socio-economic indexes for areas
- Transport/access to transport

## Social Attitudes, Beliefs, Perceptions, Political Views

- Perceived importance of social/political issues
- Perceived social status
- Social attitudes and beliefs in relation to:
  - Ageing/ageism/age in relation to work/retirement and government benefits
  - Arts/culture and their relationship to health
  - Australian history
  - Australian indigenous/Aboriginal and Torres Strait Islander people
  - Buying local goods/products
  - Caring and volunteering
  - Charities
  - Childhood/childcare
  - Climate change
  - Drug use
  - Drug regulation
  - Education and health care
  - Environmental issues
  - Ethnicity
  - Family and changing gender roles
  - Food waste
  - Genetic testing
  - Global uncertainty
  - Government and policy (confidence in government agencies, views on social security payments)
  - Government responses and rights in relation to epidemics and pandemics including the COVID-19 pandemic
  - Health/healthcare/public health
  - Immigration
  - Insurance and insurance companies
  - Kindness/prosociality
  - Law and authority
  - Pill testing
  - Present/future social challenges
  - Sexuality/sexual orientation
  - Social policies (e.g., views on who should provide care to the sick/elderly, income inequality, social benefits)
  - Social relationships
  - STIs
  - Work
- Perceptions and experiences related to health care
- Religiosity
- Political beliefs and behaviour (e.g., voting, campaigning)
- Perceptions and experiences in relation to work (including health in relation to work, job satisfaction)
- Perceived challenges of COVID-19
- Knowledge of the North and South Poles and Antarctic research
- Connection to nature
- Perceptions of community leadership and change
- Trust in others, trust in institutions

## Disability

- Disability status
- Impact of disability
- Severity of disability
- Use of self-care/mobility aids and equipment
- Need for assistance with activities of daily living
- Receipt of assistance with activities of daily living
- Assistance provided by carer
- Community participation
- NDIS participation
- Feelings of safety at home
- Service utilisation experiences
- Accessibility
- Disability-related discrimination

## Caring/Caregiving/Household Responsibilities

- Household responsibilities
- Caring/Caregiving responsibilities
- Impact of caring responsibilities
- Days off work/study due to caring responsibilities
- Work and Family Commitments
- Use of child care

## Personality

- Optimism
- Adaptability
- Self-efficacy/Perceived ability to work toward goals
- Empathy
- Agreeableness
- Conscientiousness
- Neuroticism
- Extraversion
- Intellect/Openness
- Perceived control of life events

## Physical and Biomedical Measurements

- Biomedical measures
- Physical measures (height, weight, BMI etc.)
- Blood pressure

## Technology

- Internet usage
- Electronic game usage
- Computer usage
- Main source of receiving news
- Time spent on social media
- Access to technology (computer, phone, Internet)
- Household possession of phones

## Major Life Events/Adverse Life Experiences/Victimisation

- Experiences of bullying
- Emotional abuse, neglect, physical abuse, sexual abuse, exposure to domestic violence during childhood
- Intimate partner violence
- Family stressors
- Experiences of Discrimination
- Experiences of Victimisation (e.g., physical violence/assault)
- Psychosocial events/Stressors (e.g., job loss, domestic violence, divorce)
- Stressors
- Time Stress
- Removal of respondent or family members from natural family
- Experiences of harm
- Feelings of safety at home/in community
- Victimisation/experiences of verbal and physical abuse in the context of drug or alcohol use
- Criminal justice system involvement
- Legal service utilisation
- Contact with police
- Incarceration

# Definitions of Study Name Acronyms and Abbreviations

## Table S1

*Definitions of study name acronyms and abbreviations.*

| **Acronym/Abbreviation** | **Full Study Name** |
| --- | --- |
| 45 and Up | 45 and Up Study |
| ACMS | Australian Child Maltreatment Study |
| ACWP | Australian Child Wellbeing Project |
| AHS | Australian Health Survey |
| ALSA | Australian Longitudinal Study of Ageing |
| ALSWH | Australian Longitudinal Study of Women's Health |
| ATP | Australian Temperament Project |
| AusDiab | Australian Diabetes, Obesity and Lifestyle Study (AusDiab) |
| AuSSA | Australian Survey of Social Attitudes |
| AYS | Australian Youth Survey |
| COVID MHBRCS | The Australian National COVID-19 Mental Health, Behaviour and Risk Communication Survey |
| GSS | General Social Survey |
| HILDA | The Household, Income and Labour Dynamics in Australia (HILDA) Survey |
| HOYVS | Health of Young Victorians Study |
| HWS | Health and Well-being Survey (WANTS Study) |
| IYDS | International Youth Development Survey |
| LSAC | Longitudinal Study of Australian Children |
| LSAY | Longitudinal Study of Australian Youth |
| LSAY | Longitudinal Study of Australian Youth |
| LSIC | Longitudinal Study of Indigenous Children |
| Mayi Kuwayu | Mayi Kuwayu National Study of Aboriginal and Torres Strait Islander Wellbeing |
| MCS | Middle Childhood Survey (NSW Child Development Cohort) |
| NATSIHS | National Aboriginal and Torres Strait Islander Health Survey |
| NATSISS | National Aboriginal and Torres Strait Islander Social Survey |
| NDSHS | National Drug Strategy Household survey |
| NHS | National Health Survey |
| NSMHW | National Study of Mental Health and Wellbeing |
| NSW Adult PHS | NSW Adult Population Health Survey |
| NSW Child PHS | NSW Child Population Health Survey |
| PATH Through Life | The Personality and Total Health (PATH) Through Life Study |
| SAMSS | South Australian Monitoring and Surveillance System (SAMSS) |
| SAPHS | South Australian Population Health Survey |
| SDAC | Australian Survey of Disability, Ageing and Carers (SDAC) |
| SOAR | Speak Out Against Racism (SOAR) Survey |
| SSHBS | NSW School Students Health Behaviours Survey |
| Ten to Men | Australian Longitudinal Study on Male Health (Ten to Men) |
| TTPN | Taking the Pulse of the Nation Surveys |
| VAHCS | Victorian Adolescent Health Cohort Study (VAHCS) |
| VPHS | Victorian Population Health Survey (VPHS) |
| WA HWSS | Western Australia Health and Wellbeing Surveillance System (Adult Surveys) |
| WAACHS | Western Australian Aboriginal Child Health Survey (WAACHS) |
| YMM | Australian Child and Adolescent Surveys of Mental Health and Wellbeing (Young Minds Matter) |

# Definitions of Psychological Distress Instrument Acronyms and Abbreviations

## Table S2

*Definitions of psychological distress instrument acronyms and abbreviations.*

| **Acronym/Abbreviation** | **Name of Psychological Distress Instrument** |
| --- | --- |
| CBQ | Child Behaviour Questionnaire |
| CES-D | Centre for Epidemiological Studies Depression Scale |
| CHQ | Child Health Questionnaire |
| CIDI | Composite International Diagnostic Interview |
| CIS-R | Clinical Interview Schedule – Revised |
| DASS-21 | 21-Item Depression Anxiety Stress Scale |
| DISC-IV | National Institute of Mental Health Diagnostic Interview Schedule for Children Version IV |
| DQ5 | Distress Questionnaire-5 |
| GAD-7 | 7-Item Generalized Anxiety Disorder Scale |
| GADS | Goldberg Anxiety and Depression Scale |
| GDS-SF | Geriatric Depression Scale – Short Form |
| GHQ-12 | 12-Item General Health Questionnaire |
| HBSC | Health Behaviours of School-Aged Children Study Devised Measure |
| K10 | 10-Item Kessler Psychological Distress Scale |
| K5 | 6-Item Kessler Psychological Distress Scale |
| K6 | 5-Item Kessler Psychological Distress Scale |
| MINI | Mini International Neuropsychiatric Interview |
| PedsQL | Paediatric Quality of Life Inventory |
| PHQ-9 | 9-Item Patient Health Questionnaire |
| RBPC | Revised Behaviour Problem Checklist |
| RCMAS | Revised Children’s Manifest Anxiety Scale |
| SCAS | Spence Children’s Anxiety Scale |
| SDQ | Strengths and Difficulties Questionnaire |
| SF-12 | 12-Item Short Form Health Survey/12-Item Short Form Survey/Medical Outcomes Study Health Survey Short Form (12 Items) |
| SF-36 | 36-Item Short Form Health Survey/36-Item Short Form Survey/Medical Outcomes Study Health Survey Short Form (36 Items) |
| SF-8 | 8-Item Short Form Health Survey/8-Item Short Form Survey/ /Medical Outcomes Study Health Survey Short Form (8 Items) |
| SMFQ | Short Mood and Feelings Questionnaire |
| YSR | Youth Self-Report |

# Interactive Figures

## Figure S1

*Interactive bubble plot of sample age ranges by survey year for all studies.*

Click [here](https://deannavarley.github.io/psych-distress-review-figs/interactive-bubble-plot-sample-age-range-by-study-year.html) to view the interactive plot in your browser.

## Figure S2

*Interactive bubble plot of sample age ranges by survey year for cross-sectional studies.*

Click [here](https://deannavarley.github.io/psych-distress-review-figs/interactive-bubble-plot-sample-age-range-by-study-year-cross-sectional.html)

to download and view the interactive plot in your browser.

## Figure S3

*Interactive bubble plot of sample age ranges by survey year for longitudinal studies.*

Click [here](https://deannavarley.github.io/psych-distress-review-figs/interactive-bubble-plot-sample-age-range-by-study-year-longitudinal.html) to download and view the interactive plot in your browser.

## Figure S4

*Interactive heatmap of psychological distress instrument use by study.*

Click [here](https://deannavarley.github.io/psych-distress-review-figs/interactive-heatmap-psych-distress-instrument-use-by-study.html) to download and view the interactive plot in your browser.

## Figure S5

*Interactive heatmap of other domains surveyed in each study.*

Click [here](https://deannavarley.github.io/psych-distress-review-figs/interactive-heatmap-additional-domains-surveyed-by-study.html) to download and view the interactive plot in your browser.

# Additional Summary Tables

## Table S3

*Frequency with which other assessed domains were surveyed in cross-sectional, longitudinal and all datasets.*

| **Other Surveyed Domain** | **Cross-Sectional (N)** | **Cross-Sectional (%)** | **Longitudinal (N)** | **Longitudinal (%)** | **All Surveys (N)** | **All Surveys (%)** |
| --- | --- | --- | --- | --- | --- | --- |
| Demographics | 125 | 100.0 | 158 | 100.00 | 283 | 100.00 |
| Physical Health | 124 | 99.2 | 157 | 99.37 | 281 | 99.29 |
| Other Mental Health, Wellbeing and Cognitive Function | 109 | 87.2 | 158 | 100.00 | 267 | 94.35 |
| Alcohol and Drug Use | 98 | 78.4 | 125 | 79.11 | 223 | 78.80 |
| Social Wellbeing | 73 | 58.4 | 141 | 89.24 | 214 | 75.62 |
| Financial and Socioeconomic Information | 114 | 91.2 | 155 | 98.10 | 269 | 95.05 |
| Social Attitudes | 46 | 36.8 | 86 | 54.43 | 132 | 46.64 |
| Disability | 48 | 38.4 | 119 | 75.32 | 167 | 59.01 |
| Caring Responsibilities | 38 | 30.4 | 111 | 70.25 | 149 | 52.65 |
| Personality | 24 | 19.2 | 98 | 62.03 | 122 | 43.11 |
| Biometrics | 83 | 66.4 | 105 | 66.46 | 188 | 66.43 |
| Technology | 31 | 24.8 | 56 | 35.44 | 87 | 30.74 |
| Major or Adverse Life Events | 50 | 40.0 | 123 | 77.85 | 173 | 61.13 |

## Table S4

*Psychological distress instrument usage in cross-sectional, longitudinal and all surveys as a percentage of total number of measurements of psychological distress.*

| **Instrument** | **Uses in Cross-Sectional Surveys (N)** | **% of Measurements of Psychological Distress in Cross-Sectional Surveys Using Instrument** | **Uses in Longitudinal Surveys (N)** | **% of Measurements of Psychological Distress in Longitudinal Surveys Using Instrument** | **Uses in All Surveys (N)** | **% of Measurements of Psychological Distress in All Surveys Using Instrument** |
| --- | --- | --- | --- | --- | --- | --- |
| K10 | 88 | 55.70 | 26 | 8.61 | 114 | 24.78 |
| K6 | 5 | 3.16 | 11 | 3.64 | 16 | 3.48 |
| K5 | 5 | 3.16 | 4 | 1.32 | 9 | 1.96 |
| SF-8 | 16 | 10.13 | 0 | 0.00 | 16 | 3.48 |
| SF-12 | 6 | 3.80 | 13 | 4.30 | 19 | 4.13 |
| SF-36 | 4 | 2.53 | 59 | 19.54 | 63 | 13.70 |
| SDQ | 8 | 5.06 | 8 | 2.65 | 16 | 3.48 |
| CHQ | 1 | 0.63 | 0 | 0.00 | 1 | 0.22 |
| CIDI | 3 | 1.90 | 5 | 1.66 | 8 | 1.74 |
| DISC-IV | 1 | 0.63 | 0 | 0.00 | 1 | 0.22 |
| DASS-21 | 0 | 0.00 | 8 | 2.65 | 8 | 1.74 |
| YSR | 2 | 1.27 | 0 | 0.00 | 2 | 0.43 |
| CES-D | 1 | 0.63 | 26 | 8.61 | 27 | 5.87 |
| MINI | 1 | 0.63 | 0 | 0.00 | 1 | 0.22 |
| GADS | 0 | 0.00 | 29 | 9.60 | 29 | 6.30 |
| PHQ-9 | 0 | 0.00 | 21 | 6.95 | 21 | 4.57 |
| GAD-7 | 0 | 0.00 | 10 | 3.31 | 10 | 2.17 |
| PedsQL | 0 | 0.00 | 3 | 0.99 | 3 | 0.65 |
| SCAS | 0 | 0.00 | 2 | 0.66 | 2 | 0.43 |
| GHQ-12 | 1 | 0.63 | 10 | 3.31 | 11 | 2.39 |
| SMFQ | 0 | 0.00 | 11 | 3.64 | 11 | 2.39 |
| DQ5 | 0 | 0.00 | 8 | 2.65 | 8 | 1.74 |
| HBSC | 1 | 0.63 | 0 | 0.00 | 1 | 0.22 |
| CBQ | 0 | 0.00 | 2 | 0.66 | 2 | 0.43 |
| RBPC | 0 | 0.00 | 3 | 0.99 | 3 | 0.65 |
| RCMAS | 0 | 0.00 | 2 | 0.66 | 2 | 0.43 |
| GDS-SF | 0 | 0.00 | 1 | 0.33 | 1 | 0.22 |
| CIS-R | 0 | 0.00 | 7 | 2.32 | 7 | 1.52 |
| Other Instrument | 15 | 9.49 | 33 | 10.93 | 48 | 10.43 |

## Table S5

*Psychological distress instrument usage in cross-sectional, longitudinal and all surveys. Percentages reflect the proportion of surveys to use a given instrument.*

| **Instrument** | **Uses in Cross-Sectional Surveys (N)** | **% of Cross-Sectional Surveys Using Instrument** | **Uses in Longitudinal Surveys (N)** | **% of Longitudinal Surveys Using Instrument** | **Uses in All Surveys (N)** | **% of All Surveys Using Instrument** |
| --- | --- | --- | --- | --- | --- | --- |
| K10 | 88 | 70.4 | 26 | 16.46 | 114 | 40.28 |
| K6 | 5 | 4.0 | 11 | 6.96 | 16 | 5.65 |
| K5 | 5 | 4.0 | 4 | 2.53 | 9 | 3.18 |
| SF-8 | 16 | 12.8 | 0 | 0.00 | 16 | 5.65 |
| SF-12 | 6 | 4.8 | 13 | 8.23 | 19 | 6.71 |
| SF-36 | 4 | 3.2 | 59 | 37.34 | 63 | 22.26 |
| SDQ | 8 | 6.4 | 8 | 5.06 | 16 | 5.65 |
| CHQ | 1 | 0.8 | 0 | 0.00 | 1 | 0.35 |
| CIDI | 3 | 2.4 | 5 | 3.16 | 8 | 2.83 |
| DISC-IV | 1 | 0.8 | 0 | 0.00 | 1 | 0.35 |
| DASS-21 | 0 | 0.0 | 8 | 5.06 | 8 | 2.83 |
| YSR | 2 | 1.6 | 0 | 0.00 | 2 | 0.71 |
| CES-D | 1 | 0.8 | 26 | 16.46 | 27 | 9.54 |
| MINI | 1 | 0.8 | 0 | 0.00 | 1 | 0.35 |
| GADS | 0 | 0.0 | 29 | 18.35 | 29 | 10.25 |
| PHQ-9 | 0 | 0.0 | 21 | 13.29 | 21 | 7.42 |
| GAD-7 | 0 | 0.0 | 10 | 6.33 | 10 | 3.53 |
| PedsQL | 0 | 0.0 | 3 | 1.90 | 3 | 1.06 |
| SCAS | 0 | 0.0 | 2 | 1.27 | 2 | 0.71 |
| GHQ-12 | 1 | 0.8 | 10 | 6.33 | 11 | 3.89 |
| SMFQ | 0 | 0.0 | 11 | 6.96 | 11 | 3.89 |
| DQ5 | 0 | 0.0 | 8 | 5.06 | 8 | 2.83 |
| HBSC | 1 | 0.8 | 0 | 0.00 | 1 | 0.35 |
| CBQ | 0 | 0.0 | 2 | 1.27 | 2 | 0.71 |
| RBPC | 0 | 0.0 | 3 | 1.90 | 3 | 1.06 |
| RCMAS | 0 | 0.0 | 2 | 1.27 | 2 | 0.71 |
| GDS-SF | 0 | 0.0 | 1 | 0.63 | 1 | 0.35 |
| CIS-R | 0 | 0.0 | 7 | 4.43 | 7 | 2.47 |
| Other Instrument | 15 | 12.0 | 33 | 20.89 | 48 | 16.96 |

## Table S6

*Sampling designs summarised by stratification and study design.*

| **Stratification** | **Sampling Design** | **Cross-Sectional (N)** | **Cross-Sectional (%)** | **Longitudinal (N)** | **Longitudinal (%)** | **All Surveys (N)** | **All Surveys (%)** |
| --- | --- | --- | --- | --- | --- | --- | --- |
| Stratified | Census sampling | 0 | 0.00 | 0 | 0.00 | 0 | 0.00 |
|  | Quota sampling | 0 | 0.00 | 0 | 0.00 | 0 | 0.00 |
|  | Random sampling | 29 | 35.80 | 18 | 15.52 | 47 | 23.86 |
|  | Area sampling | 45 | 55.56 | 65 | 56.03 | 110 | 55.84 |
|  | Cluster sampling | 7 | 8.64 | 33 | 28.45 | 40 | 20.30 |
|  | Purposive sampling | 0 | 0.00 | 0 | 0.00 | 0 | 0.00 |
| Non-stratified | Census sampling | 1 | 2.27 | 0 | 0.00 | 1 | 1.16 |
|  | Quota sampling | 0 | 0.00 | 8 | 19.05 | 8 | 9.30 |
|  | Random sampling | 8 | 18.18 | 27 | 64.29 | 35 | 40.70 |
|  | Area sampling | 35 | 79.55 | 0 | 0.00 | 35 | 40.70 |
|  | Cluster sampling | 0 | 0.00 | 2 | 4.76 | 2 | 2.33 |
|  | Purposive sampling | 0 | 0.00 | 5 | 11.90 | 5 | 5.81 |

## Table S7

*Use of stratification summarised by study design.*

| **Stratification** | **Cross-Sectional (N)** | **Cross-Sectional (%)** | **Longitudinal (N)** | **Longitudinal (%)** | **All Surveys (N)** | **All Surveys (%)** |
| --- | --- | --- | --- | --- | --- | --- |
| Non-stratified | 44 | 35.2 | 42 | 26.58 | 86 | 30.39 |
| Stratified | 81 | 64.8 | 116 | 73.42 | 197 | 69.61 |

## Table S8

*Sampling design summarised by study design.*

| **Sampling Design** | **Cross-Sectional (N)** | **Cross-Sectional (%)** | **Longitudinal (N)** | **Longitudinal (%)** | **All Surveys (N)** | **All Surveys (%)** |
| --- | --- | --- | --- | --- | --- | --- |
| Stratified area sampling | 45 | 36.0 | 65 | 41.14 | 110 | 38.87 |
| Stratified random sampling | 29 | 23.2 | 18 | 11.39 | 47 | 16.61 |
| Stratified cluster sampling | 7 | 5.6 | 33 | 20.89 | 40 | 14.13 |
| Non-stratified random sampling | 8 | 6.4 | 27 | 17.09 | 35 | 12.37 |
| Non-stratified area sampling | 35 | 28.0 | 0 | 0.00 | 35 | 12.37 |
| Quota sampling | 0 | 0.0 | 8 | 5.06 | 8 | 2.83 |
| Non-stratified, non-random purposive sampling | 0 | 0.0 | 5 | 3.16 | 5 | 1.77 |
| Non-stratified cluster sampling | 0 | 0.0 | 2 | 1.27 | 2 | 0.71 |
| Census sampling | 1 | 0.8 | 0 | 0.00 | 1 | 0.35 |

## Table S9

*Detailed summary of population represented summarised by study design and level of representation (state/national).*

| **Populations Represented on a National Level** | **Cross-Sectional (N)** | **Cross-Sectional (%)** | **Longitudinal (N)** | **Longitudinal (%)** | **All Surveys (N)** | **All Surveys (%)** |
| --- | --- | --- | --- | --- | --- | --- |
| Aboriginal and Torres Strait Islander people | 5 | 11.90 | 0 | 0.00 | 5 | 3.40 |
| Adolescents and Adults | 5 | 11.90 | 4 | 3.81 | 9 | 6.12 |
| Adults | 23 | 54.76 | 31 | 29.52 | 54 | 36.73 |
| Children and Adolescents | 3 | 7.14 | 0 | 0.00 | 3 | 2.04 |
| People aged 60 years and over, people with disabilities and their carers | 6 | 14.29 | 0 | 0.00 | 6 | 4.08 |
| Aboriginal and Torres Strait Islander children (at time of recruitment) | 0 | 0.00 | 5 | 4.76 | 5 | 3.40 |
| Aboriginal and Torres Strait Islander people over the age of 16 | 0 | 0.00 | 2 | 1.90 | 2 | 1.36 |
| Adolescents | 0 | 0.00 | 4 | 3.81 | 4 | 2.72 |
| Adolescents (at time of recruitment) | 0 | 0.00 | 13 | 12.38 | 13 | 8.84 |
| Australian boys and men | 0 | 0.00 | 4 | 3.81 | 4 | 2.72 |
| Australian women born 1921-1926 | 0 | 0.00 | 7 | 6.67 | 7 | 4.76 |
| Australian women born 1946-1951 | 0 | 0.00 | 10 | 9.52 | 10 | 6.80 |
| Australian women born 1973-1978 | 0 | 0.00 | 9 | 8.57 | 9 | 6.12 |
| Australian women born 1989-1995 | 0 | 0.00 | 6 | 5.71 | 6 | 4.08 |
| Children | 0 | 0.00 | 10 | 9.52 | 10 | 6.80 |
|  |  |  |  |  |  |  |
| **Populations Represented on a State Level** | **Cross-Sectional (N)** | **Cross-Sectional (%)** | **Longitudinal (N)** | **Longitudinal (%)** | **All Surveys (N)** | **All Surveys (%)** |
| Aboriginal and Torres Strait Islander children and adolescents | 1 | 1.20 | 0 | 0.00 | 1 | 0.74 |
| Adolescents and Adults | 22 | 26.51 | 0 | 0.00 | 22 | 16.18 |
| Adults | 43 | 51.81 | 15 | 28.30 | 58 | 42.65 |
| Children | 1 | 1.20 | 0 | 0.00 | 1 | 0.74 |
| Children and Adolescents | 11 | 13.25 | 6 | 11.32 | 17 | 12.50 |
| Children, Adolescents and Adults | 5 | 6.02 | 0 | 0.00 | 5 | 3.68 |
| Adolescents (at time of recruitment) | 0 | 0.00 | 11 | 20.75 | 11 | 8.09 |
| Adults aged over 45 | 0 | 0.00 | 3 | 5.66 | 3 | 2.21 |
| Adults aged over 70 | 0 | 0.00 | 8 | 15.09 | 8 | 5.88 |
| Children (at time of recruitment) | 0 | 0.00 | 10 | 18.87 | 10 | 7.35 |

## Table S10

*Survey representativeness type (national/state) summarised by study design.*

| **Study Type** | **Representativeness** | **Count** | **Percentage (%)** |
| --- | --- | --- | --- |
| Cross-sectional | National | 42 | 33.60 |
| Cross-sectional | State | 83 | 66.40 |
| Longitudinal | National | 105 | 66.46 |
| Longitudinal | State | 53 | 33.54 |
| All surveys | National | 147 | 51.94 |
| All surveys | State | 136 | 48.06 |

## Table S11

*Survey region summarised by study design.*

| **Region** | **Cross-Sectional (N)** | **Cross-Sectional (%)** | **Longitudinal (N)** | **Longitudinal (%)** | **All Surveys (N)** | **All Surveys (%)** |
| --- | --- | --- | --- | --- | --- | --- |
| National | 42 | 33.6 | 105 | 66.46 | 147 | 51.94 |
| VIC | 21 | 16.8 | 27 | 17.09 | 48 | 16.96 |
| NSW | 32 | 25.6 | 3 | 1.90 | 35 | 12.37 |
| WA | 22 | 17.6 | 0 | 0.00 | 22 | 7.77 |
| ACT | 0 | 0.0 | 15 | 9.49 | 15 | 5.30 |
| SA | 6 | 4.8 | 8 | 5.06 | 14 | 4.95 |
| NSW and VIC | 1 | 0.8 | 0 | 0.00 | 1 | 0.35 |
| WA, NT, SA | 1 | 0.8 | 0 | 0.00 | 1 | 0.35 |

# Characteristics of Psychological Distress Scales

## Table S12

*Key characteristics and summary information for each psychological distress scale identified in this scoping review, including brief details of studies evaluating the reliability and validity of each instrument in Australian samples.*

| **Instrument** | **Frequency of Use (*N*)** | **Purpose** | **Number of Items** | **Subscales** | **Usual Mode of Administration and Response Format** | **Reliability in Australian Samples** | **Validity in Australian Samples** |
| --- | --- | --- | --- | --- | --- | --- | --- |
| K10 [1] | 114 | Assessment of non-specific psychological distress | 10 | None | Questionnaire  5-point Likert scale ("None of the time" to "All of the time") | Shows good-excellent internal consistency in Australian samples, Cronbach’s α = .89-.90 [2–4], good test re-test reliability in both treatment-seeking and non-treatment-seeking Australian samples [5] | Discriminates between individuals with and without mental health diagnoses and corresponds well with mental health diagnoses, measures of disability and mental health service utilization in Australian (and international) samples [1, 4, 6–13] |
| SF-36 [14] | 63 | Assessment of health status | 36 | 8 subscales (physical functioning, role physical, bodily pain, general health, vitality, social functioning, role emotional, mental health). These 8 subscales are used to provide two ‘summary measures’ – one for physical health and one for mental health | Questionnaire  Binary, 3-point, 5-point, and 6-point Likert scales with several varied response scales | Shows acceptable-excellent internal consistency for all subscales in Australian samples, Cronbach’s α = 0.77-0.92 [15, 16] and moderate to high test-retest reliability for most subscales [16] | Good content and construct validity in an Australian sample; demonstrates good discrimination between people with and without health conditions, including both psychiatric and medical conditions [15] |
| GADS [17] | 29 | Detection of anxiety and depression | 18 | 2 subscales (anxiety, depression) | Questionnaire  Binary (yes/no) | Shows acceptable internal consistency in samples of Australian older adults, Cronbach's α^total scale^ = 0.81-0.84, Cronbach's α^anxiety^ = 0.74-0.77, and Cronbach's α^depression^ = 0.70-0.71 [18, 19]; reliability in general Australian samples has not been reported | Good criterion and concurrent validity in an Australian sample; shows good diagnostic effectiveness and concordance with CIDI 30-day diagnoses of generalized anxiety disorder, AUC = 0.90, 95% CI 0.86-0.93, and any depression disorder, AUC = 0.88, 95% CI 0.84-0.92 [20] |
| CES-D [21] | 27 | Measurement of depressive symptomatology in the general population | 20 | None | Questionnaire  4-point Likert (“Rarely or none of the time” to “Most or all of the time”) | Excellent internal consistency identified in sample of the general Australian adult population with Cronbach's α = 0.90 [22]. Good internal consistency in an Australian sample of women with breast or gynecologic cancer [23]. | Evidence of construct validity in samples of Australian older adults and gender sub-groups of Australian older adults [24, 25]. Evidence of the factor structure being generally invariant between genders and over time in Australian adolescents [26]. Evidence of criterion validity in an Australian sample of women with breast or gynecologic cancer [23]. |
| PHQ-9 [27] | 21 | Screener for probable depressive disorder diagnosis and assessment of severity of depressive symptoms | 9 | None | Questionnaire  4-point Likert (“Not at all” to “Nearly every day”) | Internal consistency in a sample of Australian residents with mild to moderate depressive symptoms/depression was questionable to good [28]. Internal consistency in sample of Australian coronary artery disease patients was excellent [29]. Good internal consistency identified for the PHQ-9 in a sample of Australian heart transplant recipients [4] and for an adapted form of the PHQ-9 in a sample of Aboriginal and Torres Strait Islander people with ischemic heart disease [30] | High concordance with CIDI 30-day depression diagnoses, with good discrimination between cases and non-cases in a sample of Australian adults [20]. Good criterion validity in a sample of Australian coronary artery disease patients [29]. Adapted forms of the PHQ-9 had acceptable performance as a screening measure for depression in a sample of Aboriginal and Torres Strait Islander Australians [30–32]. Good construct and criterion validity of the PHQ-9 identified in a sample of Australian heart transplant recipients, with good sensitivity (0.86) and specificity (0.93) [4]. |
| SF-12 [33] | 19 | Assessment of health status | 12 | 8 subscales (physical functioning, role physical, bodily pain, general health, vitality, social functioning, role emotional, mental health) and 2 summary scores (physical health component and mental health component) | Questionnaire  Binary, 3-point Likert, 5-point Likert, 6-point Likert with several varied response scales | Good internal consistency in a sample of Australian heart and stroke patients [34] and a sample of Australian lung cancer patients [35]. We did not identify further evidence describing the reliability of the SF-12 in Australian samples. | Mixed evidence. Some studies indicate that the SF-12 may be less suitable for use in Australian samples [36] and subsamples of the Australian population such as those with cancer [35] compared to samples from other locations, or that it may be less suitable than the SF-36 [37]. However, while they didn’t broadly evaluate the validity of the SF-12, studies with both clinical and non-clinical samples of Australian adults have indicated that the SF-12 was an appropriate substitute for the SF-36, predicting at least  90% of the variance in its’ physical and mental component summary scales [38] and correlating almost perfectly with the SF-36 [39]. Other studies provide evidence of construct validity in a sample of Australian heart and stroke patients [34]and discriminative validity and responsiveness to change over time for chronic disease conditions in a sample of Australian private health insurance members [40] |
| SF-8 [41] | 16 | Assessment of health status | 8 | Single-item assessment of 8 domains (physical functioning, role physical, bodily pain, general health, vitality, social functioning, role emotional, mental health) and 2 summary scores (physical health component and mental health component) | Questionnaire  5-point Likert, 6-point Likert with several varied response scales | We did not identify evidence describing the reliability of the SF-8 in Australian samples. | We did not identify evidence describing the validity of the SF-8 in Australian samples. |
| SDQ [42] | 16 | Brief assessment of behaviours, emotions, and relationships in children and adolescents | 25 | 5 subscales (emotional symptoms, conduct problems, hyperactivity/inattention, peer relationship problems, prosocial behaviour). All subscales other than prosocial behaviour can be summed to create a ‘total difficulties’ summary measure | Questionnaire  3-point Likert scale (“Not true” to “Certainly true”) | To our knowledge, there is no published information available about the reliability of the youth-report version of the SDQ in Australian samples. Parent-reported SDQ data for a sample of Australian children aged 4-9 shows good internal consistency for the total difficulties summary measure, Cronbach’s α = 0.82, acceptable to good internal consistency for the hyperactivity/inattention and prosocial behaviour subscales, Cronbach’s α = 0.80 and 0.70, but questionable internal consistency for the emotional symptoms and conduct problems subscales, Cronbach’s α = 0.66 and 0.66, and poor internal consistency for the peer relationship problems subscale, Cronbach’s α = 0.59; however, internal consistency of the assessments of test-retest reliability of over 12 months show fair stability in scores, with *r*s = 0.61-0.77 (Hawes & Dadds, 2004) ; parent-reported SDQ data for a sample of Aboriginal and/or Torres Strait Islander children aged 4-17 shows good internal consistency for the total difficulties summary measure, Cronbach’s α = 0.85, and acceptable internal consistency for the emotional symptoms subscale, α = 0.70, conduct problems subscale, α = 0.78, hyperactivity/inattention subscale, α = 0.79, and prosocial behaviour subscale, α = 0.78. However, the internal consistency for the peer relationship problems subscale was poor, α = 0.47 (Williamson et al., 2014) | The parent-report, teacher-report, and youth-report versions of the SDQ all showed reasonable clinical validity in a clinical sample of Australian children aged 4-14 admitted to a Child and Adolescent Mental Health Service, with moderate correlations between the total difficulties summary measures and clinician-rated Health of the Nation Outcome Scales for Children and Adolescents (Mathai et al., 2002). Parent-reported SDQ data for a sample of Australian children aged 4-9 shows good criterion validity, concurrent validity, and predictive validity; provides good prediction of DSM-IV diagnoses, clinician-rated severity of related diagnostic features, and receipt of treatment for emotional/behavioural problems (Hawes & Dadds, 2004); parent-reported SDQ data for a sample of Aboriginal and/or Torres Strait Islander children aged 4-17 shows acceptable construct validity and good convergent validity, with the total difficulties summary measure corresponding well with separate parent-reports of problematic behaviours in their children in the last 6 months (Williamson et al., 2014) |
| K6 [1, 43] | 16 | Assessment of non-specific psychological distress | 6 | None | Questionnaire  5-point Likert scale ("None of the time" to "All of the time") | Shows excellent internal consistency in a sample of Australian adults, Cronbach’s α = .93 [2], and good internal consistency in Australian adolescents, Cronbach’s α = .84 [44] | Good criterion and concurrent validity; provides good prediction of mental health diagnoses and serious impairment associated with DSM-IV mood, anxiety and substance use disorders in Australian adults [8, 12], corresponds well with scores on the SDQ for Australian adolescent women, but had less predictive utility for adolescent men [44]. Acceptable discriminant validity and good sensitivity and specificity when tested in a sample of Australian residents receiving Internet-delivered cognitive behavioural therapy (sensitivity = 0.68, specificity = 0.69) [45] |
| SMFQ [46] | 11 | Screener for probable depressive disorder diagnosis/symptoms of depression in children and adolescents, designed for use in epidemiological studies | 13 | None | Questionnaire  3-point Likert (“True” to “Not true”) | We did not identify evidence of the reliability of the SMFQ when tested in Australian samples. | We did not identify evidence of the validity of the SMFQ when tested in Australian samples. |
| GHQ-12 [47, 48] | 11 | Brief screener to identify those at risk of developing psychiatric disorders | 12 | None | Questionnaire  4-point Likert (“Less than usual” to “Much more than usual”) | Good internal consistency in samples of Australian adults, Cronbach’s α = 0.85, Australian adolescents, Cronbach’s α = 0.86-0.89, and Australian children, Cronbach’s α = 0.82-0.88, however, some evidence of some items (8 and 11) being less reliable in adolescents [49–52] | Mixed evidence. A review noted the GHQ-12 may be less suitable for use in samples of Australian adults than samples of adults from other countries, with sensitivity of 75.4% and specificity of 69.9%. Likewise, a study suggested criterion validity may be poorer in Australian older adults than samples from other countries. Other studies provide evidence of its convergent validity and construct validity with evidence of measurement invariance and good fit of the same factor structure between samples of Australian adolescents and Australian adults, and, in a separate study, between Australian children and adolescents; however, best fitting factor structure varied between studies [49–53] |
| GAD-7 [54] | 10 | Brief screener to identify probable cases of generalized anxiety disorder | 7 | None | Questionnaire  4-point Likert (“Not at all” to “Nearly every day”) | Adequate to excellent internal consistency in a sample of Australian adults at two time points, Cronbach’s α = 0.79 and 0.91 [55] and excellent internal consistency in an Australian sample of heart transplant recipients, Cronbach’s α = 0.91 [4]. | Factor analysis supports a one-factor structure, aligning with intended measurement of a single construct. Some evidence of convergent validity, with moderate correlations with the Penn State Worry Questionnaire in sample of Australian adults (*r* = .51–.71), and sensitivity to change following treatment. Area under the curve (AUC) within acceptable range (0.72), with sensitivity of 78.57% and specificity of 58.99% in a sample of Australian adults with cancer. In another study with a sample of Australian adults, AUC = 0.83, sensitivity = 60.6%, specificity = 87.6% [55–57]. Acceptable sensitivity (75%) and specificity (89%) for diagnosis of an anxiety disorder (AUC = 0.82) when examined in an Australian sample of heart transplant recipients [4] |
| K5 and MK-K5 [58, 59] | 9 | Culturally sensitive assessment of non-specific psychological distress in Aboriginal and Torres Strait Islander people | 5 | None | Questionnaire  5-point Likert scale ("None of the time" to "All of the time") | Good internal consistency demonstrated in samples of Aboriginal and Torres Strait Islander people, Cronbach’s α = 0.88-0.89 [3, 58]. | Demonstrates good face validity, construct validity, convergent validity and divergent validity [3, 58]. |
| DQ5 [2] | 8 | Brief screener for measuring psychological distress and identifying probable diagnoses of common mental disorders | 5 | None | Questionnaire  5-point Likert scale (“Never” to “Always”) | Good internal consistency in a sample of Australian adults, Cronbach’s α = 0.86 [2] and excellent test-retest reliability in a sample of Australian adolescents, *r* = 0.77 [60]. | Good construct validity and good criterion validity, with good accuracy in the identification of mental health diagnoses in a sample of Australian adults [2] and strong criterion and predictive validity in a sample of Australian adolescents [60]. |
| DASS-21 [61, 62] | 8 | Measurement of three related negative emotional states: depression, anxiety, and stress | 21 | 3 subscales (depression, anxiety, stress) | Questionnaire  4-point Likert scale (“Never” to “Almost always”) | Examined in a sample of Australian adults [22], and in Australian adolescents [63, 64] | Validity evaluated in samples of Australian adults [61], Australian adolescents [63–65] and in an Australian clinical sample for its validity as a clinical outcome measure [66] |
| CIDI [67] | 8 | Diagnostic interview for assessment of mental disorders according to formal diagnostic criteria | 276 symptom questions (however, skip rules mean not all questions will be asked) with additional probe questions accompanying some items to evaluate symptom severity, help-seeking behaviour, psychosocial impairment and episodic features. | Number of sections varies across versions of the CIDI. The World Mental Health Survey Initiative version of the CIDI has 22 sections focused on diagnoses, 4 sections focused on functioning, 2 sections focused on treatment, 4 sections focused on risk factors, 7 sections focused on socio-demographic correlates, and 2 sections focused on methodological factors [68] | Interview  Response format varies. CIDI questions are structured interview questions. Some questions require yes/no responses or ask respondents to rate the severity of symptoms. | Perfect interrater reliability identified between interviewers and interview observers completing independent coding in a study with an Australian sample of adolescents and adults receiving treatment for anxiety [69] | Concordance between the CIDI and the Schedules for Clinical Assessment in Neuropsychiatry (SCAN) when examined in study with an Australian sample of adolescents and adults receiving treatment for anxiety was fair at best when measured by Intraclass Kappas = 0.34-0.62 for current and lifetime diagnoses of four mental health diagnoses (Depression, Agoraphobia, Social Phobia, Obsessive Compulsive Disorder), and moderate when measured by canonical correlation analysis for current (*r* = 0.69) and lifetime (*r* = 0.66) diagnoses [69]. A study with an Australian sample of outpatients with suspected anxiety or depressive disorders identified poor agreement between experienced clinicians and a computerized version of the CIDI [70]. However, another study with patients accepts for treatment at an Australian anxiety disorders clinic indicated that a computerized version of the CIDI identified 88.2% of clinician-made diagnoses but also identified twice as many diagnoses as clinicians. Compared to diagnoses made using longitudinal data, expert consensus and all available patient data, the sensitivity of the CIDI for clinical diagnoses made was >85% for six anxiety and mood diagnoses, except for Generalized Anxiety Disorder, for which sensitivity was 29%. The specificity ranged widely between 47-99%. Agreement between CIDI and clinician diagnoses measured by intraclass Kappas also ranged widely from poor (*k* = 0.02 for GAD) to excellent (*k* = 0.81; OCD), but with overall agreement being fair (*k* = 0.40). Overall, results suggested the CIDI may provide diagnoses at a lower threshold compared to experienced clinicians [71]. Concordance between the CIDI and DSM-IV diagnoses made by clinicians after a standard semi-structured clinical interview measured via Kappa varied between less than chance agreement to moderate agreement in an urban Aboriginal and Torres Strait Islander sample [72]. Concordance between the CIDI and DSM-IV diagnoses made by clinical nurse consultants or registrar or consultant psychiatrists in a sample of Australian adolescent and adult deliberate self-poisoning patients was poor across diagnoses for anxiety, depressive and substance-use disorders [73]. |
| CIS-R [74] | 7 | Standardised assessment of common mental disorders | Varies according to answers provided by respondent | 14 subsections (somatic symptoms, fatigue, concentration and forgetfulness, sleep problems, irritability, worry about physical health, depression, depressive ideas, worry, anxiety, phobias, panic, compulsions, obsessions) and an additional section that assesses the overall effect of symptoms assessed in the first 14 sections | Interview  Response format varies. CIS-R questions are structured interview questions. Some questions are close-ended questions requiring yes/no responses while others (e.g., those related to symptom frequency) require responses on scales or ask respondents to rate the severity of symptoms. | We did not identify evidence of the reliability of the CIS-R when tested in Australian samples. | Evidence for criterion validity in a sample of Australian adolescents, with the CIS-R showing high specificity (0.97) and NPV (0.91), but low sensitivity (0.18) and moderate PPV (0.49) for CIDI diagnoses of depression in the past 6 months, indicating good ability to rule out cases but limited ability to detect true positives [75] |
| PedsQL [76] | 3 | Assessment of health-related quality of life in children and adolescents | 23 | 4 subscales (physical functioning, emotional functioning, social functioning, school functioning) | Questionnaire  5-point Likert scale (“Never” to “Almost always”) | Demonstrates acceptable test-retest reliability in an Australian sample of children and adolescents [77] | Demonstrates known-group validity, convergent validity, divergent validity and responsiveness to worsening health in a sample of Australian children [77] |
| RBPC (self-reported) [78] | 3 | Assessment of problematic behaviours in children | 89 | 6 subscales (conduct disorder, socialized aggression, attention problems/immaturity, anxiety/withdrawal, psychotic behaviour) | Questionnaire  3-point Likert (“No problem” to “Severe problem”) | We did not identify evidence of the reliability of the RBPC as a self-report measure tested in Australian samples. | We did not identify evidence of the validity of the RBPC as a self-report measure tested in Australian samples. |
| YSR [79, 80] | 2 | Measurement of emotional/behavioural problems and social competencies in children and adolescents | 112 (plus additional open-ended questions) | 2 competence subscales (social competence, activities) and 8 syndrome subscales (withdrawn/depressed, somatic complaints, anxious/depressed, social problems, thought problems, attention problems, rule-breaking behaviour, and aggressive behaviour). Also provides summary scores, including two “broadband scales” assessing internalising and externalising behaviours, a ‘total problems’ summary score, and a ‘total competence’ summary score. | Questionnaire  3-point Likert (“Not true” to “Very true or often true”) and open-ended questions | Limited evaluations of the reliability of the YSR in Australian samples. A study in a sample of Australian adolescent psychiatric outpatients suggested that cross-informant agreement between parents and adolescents was comparable to levels of agreement between teachers and parents for externalizing behaviors but cross-informant agreement between parents and adolescents was poorer compared to agreement between teachers and parents for internalizing behaviors [81]. | Some evidence for convergent and discriminant validity when examined in a sample of Australian adolescent psychiatric outpatients [81]. |
| SCAS [82] | 2 | Assessment of child anxiety disorders | 45 | 6 subscales (separation anxiety, social phobia, obsessive compulsive, panic/agoraphobia, physical injury fears, generalised anxiety) | Questionnaire  4-point Likert (“Never” to “Always”) | When examined in a sample of Australian children, internal consistency was excellent for the total scale (Cronbach’s α = 0.92) and varied between questionable to good for the subscales (Cronbach’s αs = 0.60-0.82) and test-retest reliability correlation coefficients were *r =* 0.60 for the total scale and ranged between 0.45-0.57 for the subscales [83]. Similar results identified in a sample of Australian adolescents [84]. | Confirmatory factor analyses in a sample of Australian school children provided support for a factor structure proposed a priori aligned with DSM-IV diagnostic categories [82]. Evidence of convergent and discriminant validity when examined in a sample of Australian children [83]. Similar results identified in a sample of Australian adolescents [84]. |
| CBQ (self-reported) [85, 86] | 2 | Assessment of behavioural disturbances in children | 26-31 | 3 subscales (conduct disorder, emotional disorder, and hyperactivity) | Questionnaire  3-point Likert (“Does not apply” to “Certainly applies”) | We did not identify evidence of the reliability of the CBQ as a self-report measure tested in Australian samples. | Some evidence of diagnostic concordance between child self-report via the CBQ and DSM-III-R diagnoses via structured psychiatric interview with a psychologist using the Child Assessment Schedule when examined in a sample of Australian children, with DSM-III-R diagnoses identified by CBQ subscales ranging between 61-67% [86]. |
| RCMAS [87] | 2 | Measurement of manifest anxiety in children | 37 | 3 subscales (physiological anxiety, worry-sensitivity, and social concerns-concentration) and an additional social desirability/lie scale | Questionnaire  Binary (yes/no) | Good internal consistency in samples of Australian children and adolescents (Cronbach’s α = 0.81-0.87) [88–91]. A version of the RCMAS adapted for use with children with intellectual disabilities demonstrated good internal consistency when tested in an Australian sample of children with intellectual disabilities (Cronbach’s α = 0.88) [92] | Evidence of convergent validity in samples of Australian children and adolescents [88, 89, 91, 93]. Some evidence of convergent validity of a version of the RCMAS adapted for use with children with intellectual disabilities when examined in an Australian sample of children with intellectual disabilities [92] |
| CHQ (self-reported) [94] | 1 | Measurement of functional health status and wellbeing | 87 (80 in an updated version), also available as a 45 item short-form | 12 multi-item subscales (physical functioning, role social-emotional, role social-behavioural, role social-physical, bodily pain, behaviour, mental health, self-esteem, general health, family activities) and 2 single-item subscales (family cohesion, change in health) | Questionnaire  4-point Likert, 5-point Likert, 6-point Likert with several varied response scales | Internal consistency good to excellent across subscales in a sample of Australian adolescents (Cronbach’s αs = 0.75-0.90) [95] | Evidence of construct validity in a sample of Australian sample of adolescents [95] |
| MINI [96, 97] | 1 | Diagnosis of mental disorders according to DSM and/or ICD criteria | Varies according to answers provided by respondent | Provides assessment of the presence of 17 disorders (major depressive disorder, dysthymic disorder, suicidality, mania, panic disorder, agoraphobia, social phobia, specific phobia, obsessive-compulsive disorder, generalised anxiety disorder, alcohol dependence, alcohol abuse, drug dependence (non-alcohol), drug abuse (non-alcohol), psychotic disorder, anorexia nervosa, bulimia, posttraumatic stress disorder, antisocial personality disorder) | Interview  Close-ended questions (i.e., yes/no questions) | We did not identify evidence of the reliability of the MINI tested in Australian samples. | We did not identify evidence of the validity of the MINI being directly tested in Australian samples. However, it should be noted that studies evaluating the validity of other instruments in Australian samples have reviewed whether those instruments are associated with the MINI in the expected direction/have tested the validity of other instruments while using the MINI as the criterion standard [4, 13, 23, 29, 32, 45, 57, 98–103]. |
| HBSC-SCL [104] | 1 | Measurement of psychosomatic health as an indicator of mental health in adolescence, involving assessment of prevalence of eight psychosomatic complaints common in adolescents including headache, stomachache, backache, feeling low, irritability/bad temper, feeling nervous, difficulties in getting to sleep and feeling dizzy | 8 (however, some versions of this scale have additional items) | None | Questionnaire  5-point Likert (“about every day” to “about every month: rarely or never”) | We did not identify evidence of the reliability of the HBSC tested in Australian samples. | We did not identify evidence of the validity of the HBSC tested in Australian samples. |
| GDS-SF [105] | 1 | Screener for depression in older adults including those with mild to moderate dementia and physical illness | 15 | None | Questionnaire  Binary (yes/no) | Good internal consistency in an Australian sample of older adults residing in aged care facilities, Cronbach’s α = 0.83 [106] | Some evidence of associations with related constructs in expected directions in an Australian sample of older adults residing in aged care facilities [106]. Additionally, while the short form was not evaluated, a study in an Australian sample of stroke patients identified the longer form of the GDS as a satisfactory screening instrument for depression [107]. Similarly, the long form GDS has been identified to have a Using 11 as a cutoff point, the GDS was found to have 93% sensitivity and 83% specificity for identifying DSM-III affective disorders in an Australian sample of aged care residents [108]. |

# References for Supplementary Document

1. Kessler RC, Andrews G, Colpe LJ, Hiripi E, Mroczek DK, Normand SLT, Walters EE, Zaslavsky AM (2002) Short screening scales to monitor population prevalences and trends in non-specific psychological distress. Psychol Med 32:959–976

2. Batterham PJ, Sunderland M, Carragher N, Calear AL, Mackinnon AJ, Slade T (2016) The Distress Questionnaire-5: Population screener for psychological distress was more accurate than the K6/K10. J Clin Epidemiol 71:35–42

3. McNamara BJ, Banks E, Gubhaju L, Williamson A, Joshy G, Raphael B, Eades SJ (2014) Measuring psychological distress in older Aboriginal and Torres Strait Islanders Australians: A comparison of the K-10 and K-5. Aust N Z J Public Health 38:567–573

4. Conway A, Sheridan J, Maddicks-Law J, Fulbrook P, Ski CF, Thompson DR, Doering L V. (2016) Accuracy of anxiety and depression screening tools in heart transplant recipients. Applied Nursing Research 32:177–181

5. Merson F, Newby J, Shires A, Millard M, Mahoney A (2021) The temporal stability of the Kessler Psychological Distress Scale. Aust Psychol 56:38–45

6. Andrews G, Slade T (2001) Interpreting scores on the Kessler Psychological Distress Scale (K10). Aust N Z J Public Health 25:494–497

7. Fassaert T, De Wit MAS, Tuinebreijer WC, Wouters H, Verhoeff AP, Beekman ATF, Dekker J (2009) Psychometric properties of an interviewer-administered version of the Kessler Psychological Distress scale (K10) among Dutch, Moroccan and Turkish respondents. Int J Methods Psychiatr Res 18:159–168

8. Furukawa TA, Kessler RC, Slade T, Andrews G (2003) The performance of the K6 and K10 screening scales for psychological distress in the Australian National Survey of Mental Health and Well-Being. Psychol Med 33:357–362

9. Furukawa TA, Kawakami N, Saitoh M, et al (2008) The performance of the Japanese version of the K6 and K10 in the World Mental Health Survey Japan. Int J Methods Psychiatr Res 17:152–158

10. Kessler RC, Green JG, Gruber MJ, et al (2010) Screening for serious mental illness in the general population with the K6 screening scale: Results from the WHO World Mental Health (WMH) survey initiative. Int J Methods Psychiatr Res 19:4–22

11. Sampasa-Kanyinga H, Zamorski MA, Colman I (2018) The psychometric properties of the 10-item kessler psychological distress scale (K10) in canadian military personnel. PLoS One. https://doi.org/10.1371/journal. pone.0196562

12. Sunderland M, Slade T, Stewart G, Andrews G (2011) Estimating the prevalence of DSM-IV mental illness in the Australian general population using the Kessler Psychological Distress Scale. Australian and New Zealand Journal of Psychiatry 45:880–889

13. Hides L, Lubman DI, Devlin H, Cotton S, Aitken C, Gibbie T, Hellard M (2007) Reliability and validity of the Kessler 10 and Patient Health Questionnaire among injecting drug users. Australian and New Zealand Journal of Psychiatry 41:166–168

14. Ware JEJ, Sherbourne CD (1992) The MOS 36-item short-form health survey (SF-36): I. Conceptual framework and item selection. Med Care 30:473–483

15. McCallum J (1995) The SF‐36 in an Australian sample: validating a new, generic health status measure. Aust J Public Health 19:160–166

16. Sanson-Fisher RW, Perkins JJ (1998) Adaptation and validation of the SF-36 Health Survey for use in Australia. J Clin Epidemiol 51:961–967

17. Goldberg D, Bridges K, Duncan-Jones P, Grayson D (1988) Detecting anxiety and depression in general medical settings. Br Med J 297:897–899

18. Mackinnon A, Christensen H, Jorm AF, Henderson AS, Scott R, Korten AE (1994) A latent trait analysis of an inventory designed to detect symptoms of anxiety and depression using an elderly community sample. Psychol Med 24:977–986

19. Smith N (2004) ALSWH Data Dictionary Supplement Section 2 Core Survey Dataset 2.7 Psychosocial Variables - Goldberg Anxiety and Depression Inventory (GADS).

20. Kiely KM, Butterworth P (2015) Validation of four measures of mental health against depression and generalized anxiety in a community based sample. Psychiatry Res 225:291–298

21. Radloff LS (1977) The CES-D scale: A self-report depression scale for research in the general population. Appl Psychol Meas 1:385–401

22. Crawford J, Cayley C, Lovibond PF, Wilson PH, Hartley C (2011) Percentile norms and accompanying interval estimates from an Australian general adult population sample for self-report mood scales (BAI, BDI, CRSD, CES-D, DASS, DASS-21, STAI-X, STAI-Y, SRDS, and SRAS). Aust Psychol 46:3–14

23. Stafford L, Judd F, Gibson P, Komiti A, Quinn M, Mann GB (2014) Comparison of the Hospital Anxiety and Depression Scale and the Center for Epidemiological Studies Depression Scale for detecting depression in women with breast or gynecologic cancer. Gen Hosp Psychiatry 36:74–80

24. Mohebbi M, Nguyen V, McNeil JJ, et al (2018) Psychometric properties of a short form of the Center for Epidemiologic Studies Depression (CES-D-10) scale for screening depressive symptoms in healthy community dwelling older adults. Gen Hosp Psychiatry 51:118–125

25. McCallum J, Mackinnon A, Simons L, Simons J (1995) Measurement properties of the Center for Epidemiological Studies Depression Scale: An Australian community study of aged persons. Journal of Gerontology: Series B 50B:182–189

26. Verhoeven M, Sawyer MG, Spence SH (2013) The factorial invariance of the CES-D during adolescence: Are symptom profiles for depression stable across gender and time? J Adolesc 36:181–190

27. Kroenke K, Spitzer RL, Williams JBW (2001) The PHQ-9: Validity of a brief depression severity measure. J Gen Intern Med 16:606–613

28. Titov N, Dear BF, McMillan D, Anderson T, Zou J, Sunderland M (2011) Psychometric comparison of the PHQ-9 and BDI-II for measuring response during treatment of depression. Cogn Behav Ther 40:126–136

29. Stafford L, Berk M, Jackson HJ (2007) Validity of the Hospital Anxiety and Depression Scale and Patient Health Questionnaire-9 to screen for depression in patients with coronary artery disease. Gen Hosp Psychiatry 29:417–424

30. Esler D, Johnston F, Thomas D, Davis B (2008) The validity of a depression screening tool modified for use with Aboriginal and Torres Strait Islander people. Aust N Z J Public Health 32:317–321

31. Hackett ML, Teixeira-Pinto A, Farnbach S, Glozier N, Skinner T, Askew DA, Gee G, Cass A, Brown A (2019) Getting it right: Validating a culturally specific screening tool for depression (aPHQ-9) in Aboriginal and Torres Strait Islander Australians. Medical Journal of Australia 211:24–30

32. Skinner T, Brown A, Teixeira-Pinto A, Farnbach SF, Glozier N, Askew DA, Gee G, Cass A, Hackett ML (2024) Sensitivity and specificity of Aboriginal-developed items to supplement the adapted PHQ-9 screening measure for depression: Results from the Getting it Right study. Medical Journal of Australia 221:258–263

33. Ware J, Kosinski M, Keller S (1996) A 12-Item Short Form Health Survey: Construction of scales and preliminary tests of reliability and validity. Med Care 34:220–233

34. Lim LLY, Fisher JD (1999) Use of the 12-item Short-Form (SF-12) Health Survey in an Australian heart and stroke population. Quality of Life Research 8:1–8

35. Soh SE, Morello R, Ayton D, Ahern S, Scarborough R, Zammit C, Brand M, Stirling RG, Zalcberg J (2021) Measurement properties of the 12-item Short Form Health Survey version 2 in Australians with lung cancer: A Rasch analysis. Health Qual Life Outcomes. https://doi.org/10.1186/s12955-021-01794-w

36. McCallum J (1997) The shorter short form: Analysis of SF-12 items in Australian data. Integrating Health Outcomes Measurement in Routine Health Care

37. Schofield MJ, Mishra G (1998) Validity of the SF-12 compared with the SF-36 Health Survey in pilot studies of the Australian Longitudinal Study on Women’s Health. J Health Psychol 3:259–271

38. Sanderson K, Andrews G (2002) The SF-12 in the Australian population: Cross-validation of item selection. Aust N Z J Public Health 26:343–345

39. Andrews G (2002) A brief integer scorer for the SF-12: Validity of the brief scorer in Australian community and clinic settings. Aust N Z J Public Health 26:508–510

40. Le Grande MR, Tucker G, Bunker S, Jackson AC (2019) Validating the Short Form-12 and the development of disease-specific norms in a cohort of Australian private health insurance members. Aust J Prim Health 25:90–96

41. Ware JE, Kosinski M, Dewey JE, Gandek B (2001) How to score and interpret single-item health status measures: a manual for users of the SF-8 health survey. QualityMetric Incorporated, Lincoln, Rhode Island

42. Goodman R (1997) The strengths and difficulties questionnaire: A research note. J Child Psychol Psychiatry 38:581–586

43. Kessler RC, Barker PR, Colpe LJ, et al (2003) Screening for Serious Mental Illness in the General Population. Arch Gen Psychiatry 60:184–189

44. Mewton L, Kessler RC, Slade T, et al (2016) The psychometric properties of the kessler psychological distress scale (K6) in a general population sample of adolescents. Psychol Assess 28:1232–1242

45. Staples LG, Dear BF, Gandy M, Fogliati V, Fogliati R, Karin E, Nielssen O, Titov N (2019) Psychometric properties and clinical utility of brief measures of depression, anxiety, and general distress: The PHQ-2, GAD-2, and K-6. Gen Hosp Psychiatry 56:13–18

46. Angold A, Costello EJ, Messer SC, Pickles A (1995) Development of a short questionnaire for use in epidemiological studies of depression in children and adolescents. Int J Methods Psychiatr Res 5:237–249

47. Goldberg DP (1972) The detection of psychiatric illness by questionnaire: A technique for the identification and assessment of non-psychotic psychiatric illness. Oxford University Press

48. Goldberg D, Williams P (1988) A user’s guide to the General Health Questionnaire. GL Assessment, London

49. Tait RJ, French DJ, Hulse GK (2003) Validity and psychometric properties of the General Health Questionnaire-12 in young Australian adolescents. Australian & New Zealand Journal of Psychiatry 37:374–381

50. Winefield HR, Goldney RD, Winefield AH, Tiggemann M, Winefield HK, Goldtiey KD, Winefield AH (1989) The General Health Questionnaire: Reliability and validity for Australian youth. Australian & New Zealand Journal of Psychiatry 23:53–58

51. Centofanti S, Lushington K, Wicking A, Wicking P, Fuller A, Janz P, Dorrian J (2019) Establishing norms for mental well-being in young people (7–19 years) using the General Health Questionnaire-12. Aust J Psychol 71:117–126

52. French DJ, Tait RJ (2004) Measurement invariance in the General Health Questionnaire-12 in young Australian adolescents. Eur Child Adolesc Psychiatry 13:1–7

53. Donath S (2001) The validity of the 12-Item General Health Questionnaire in Australia: A comparison between three scoring methods. Australian & New Zealand Journal of Psychiatry 35:231–235

54. Spitzer RL, Kroenke K, Williams JBW, Löwe B (2006) A brief measure for assessing Generalized Anxiety Disorder: The GAD-7. Arch Intern Med 166:1092–1097

55. Dear BF, Titov N, Sunderland M, McMillan D, Anderson T, Lorian C, Robinson E (2011) Psychometric comparison of the Generalized Anxiety Disorder Scale-7 and the Penn State Worry Questionnaire for measuring response during treatment of Generalised Anxiety Disorder. Cogn Behav Ther 40:216–227

56. Clover K, Lambert SD, Oldmeadow C, Britton B, King MT, Mitchell AJ, Carter GL (2022) Apples to apples? Comparison of the measurement properties of hospital anxiety and depression-anxiety subscale (HADS-A), depression, anxiety and stress scale-anxiety subscale (DASS-A), and generalised anxiety disorder (GAD-7) scale in an oncology setting using Rasch analysis and diagnostic accuracy statistics. Current Psychology 41:4592–4601

57. Christensen H, Batterham PJ, Grant J, Griffiths KM, MacKinnon AJ (2011) A population study comparing screening performance of prototypes for depression and anxiety with standard scales. BMC Med Res Methodol 11:1–9

58. Brinckley MM, Calabria B, Walker J, Thurber KA, Lovett R (2021) Reliability, validity, and clinical utility of a culturally modified Kessler scale (MK-K5) in the Aboriginal and Torres Strait Islander population. BMC Public Health. https://doi.org/10.1186/s12889-021-11138-4

59. Australian Institute of Health and Welfare (2009) Measuring the social and emotional wellbeing of Aboriginal and Torres Strait Islander peoples. Canberra

60. Batterham PJ, Werner-Seidler A, O’Dea B, Calear AL, Maston K, Mackinnon A, Christensen H (2024) Psychometric properties of the Distress Questionnaire-5 (DQ5) for measuring psychological distress in adolescents. J Psychiatr Res 169:58–63

61. Lovibond SH, Lovibond PF (1995) Manual for the Depression Anxiety Stress Scales, 2nd ed. Psychology Foundation, Sydney

62. Lovibond PF, Lovibond SH (1995) The structure of negative emotional states: Comparison of the Depression Anxiety Stress Scales (DASS) with the Beck Depression and Anxiety Inventories. Behaviour Research and Therapy 33:335–343

63. Szabó M (2010) The short version of the Depression Anxiety Stress Scales (DASS-21): Factor structure in a young adolescent sample. J Adolesc 33:1–8

64. Shaw T, Campbell MA, Runions KC, Zubrick SR (2017) Properties of the DASS-21 in an Australian community adolescent population. J Clin Psychol 73:879–892

65. Mellor D, Vinet E V., Xu X, Hidayah Bt Mamat N, Richardson B, Román F (2015) Factorial invariance of the DASS-21 among adolescents in four countries. European Journal of Psychological Assessment 31:138–142

66. Ng F, Trauer T, Dodd S, Callaly T, Campbell S, Berk M (2007) The validity of the 21-item version of the Depression Anxiety Stress Scales as a routine clinical outcome measure. Acta Neuropsychiatr 19:304–310

67. World Health Organization (1990) Composite International Diagnostic Interview, Version 1.0.

68. Kessler RC, Bedirhan Üstün T (2004) The World Mental Health (WMH) Survey Initiative Version of the World Health Organization (WHO) Composite International Diagnostic Interview (CIDI). Int J Methods Psychiatr Res 13:93–121

69. Andrews G, Peters L, Guzman A-M, Bird K, Guzman Mpsychol A-M (1995) A comparison of two structured diagnostic interviews: ClDl and SCAN. Australian & New Zealand Journal of Psychiatry 29:124–132

70. Komiti AA, Jackson HJ, Judd FK, et al (2001) A comparison of the Composite International Diagnostic Interview (CIDI-Auto) with clinical assessment in diagnosing mood and anxiety disorders. Australian & New Zealand Journal of Psychiatry 35:224–230

71. Peters L, Andrews G (1995) Procedural validity of the computerized version of the Composite International Diagnostic Interview (CIDI-Auto) in the anxiety disorders. Psychol Med 25:1269–1280

72. Basit T, Anderson M, Lindstrom A, Santomauro DF, Whiteford HA, Ferrari AJ (2023) Diagnostic accuracy of the Composite International Diagnostic Interview (CIDI 3.0) in an urban Indigenous Australian sample. Australian and New Zealand Journal of Psychiatry 57:283–290

73. Jayasekera H, Carter G, Clover K (2011) Comparison of the composite international diagnostic interview (CIDI-auto) with clinical diagnosis in a suicidal population. Archives of Suicide Research 15:43–55

74. Lewis G, Pelosi AJ, Araya R, Dunn G (1992) Measuring psychiatric disorder in the community: A standardized assessment for use by lay interviewers. Psychol Med 22:465–486

75. Patton GC, Coffey C, Posterino M, Carlin JB, Wolfe R, Bowes G (1999) A computerised screening instrument for adolescent depression: Population-based validation and application to a two-phase case-control study. Soc Psychiatry Psychiatr Epidemiol 34:166–172

76. Varni JW, Seid M, Rode CA (1999) The PedsQLTM: Measurement model for the Pediatric Quality of Life Inventory. Med Care 37:126–139

77. Jones R, O’Loughlin R, Xiong X, Bahrampour M, Devlin N, Hiscock H, Chen G, Mulhern B, Dalziel K (2024) Comparative psychometric performance of common generic paediatric health-related quality of life instrument descriptive systems: Results from the Australian Paediatric Multi-Instrument Comparison Study. Pharmacoeconomics 42:39–55

78. Quay HC (1983) A dimensional approach to behavior disorder: The Revised Behavior Problem Checklist. School Psych Rev 12:244–249

79. Achenbach T (1991) Manual for the Youth Self-Report and 1991 profile. Department of Psychiatry, University of Vermont, Burlington

80. Achenbach T, Rescorla L (2001) Manual for the ASEBA school-age forms and profiles. Research Centre for Children, Youth and Families, University of Vermont, Burlington

81. Gomez R, Vance A, Gomez RM (2014) Analysis of the convergent and discriminant validity of the CBCL, TRF, and YSR in a clinic-referred sample. J Abnorm Child Psychol 42:1413–1425

82. Spence SH (1997) Structure of anxiety symptoms among children: A confirmatory factor-analytic study. J Abnorm Psychol 106:280–297

83. Spence SH (1998) A measure of anxiety symptoms among children. Behaviour Research and Therapy 36:545–566

84. Spence SH, Barrett PM, Turner CM (2003) Psychometric properties of the Spence Children’s Anxiety Scale with young adolescents. J Anxiety Disord 17:605–625

85. Rutter M, Tizzard J, Whitmore K (1970) Education, health and behaviour. Longmans, London

86. Prior M, Sanson A, Smart D, Oberklaid F (1999) Psychological disorders and their correlates in an Australian community sample of preadolescent children. Journal of Child Psychology and Psychiatry 40:563–580

87. Reynolds CR, Richmond BO (1978) What I think and feel: A revised measure of children’s manifest anxiety. Journal of Abnormal Child Psyehology 6:271–280

88. Kaczmarek EA, Sibbel AM (2008) The psychosocial well-being of children from Australian military and fly-in/fly-out (FIFO) mining families. Community Work Fam 11:297–312

89. King NJ, Gullone E, Ollendick TH (1992) Manifest anxiety and fearfulness in children and adolescents. Journal of Genetic Psychology 153:63–73

90. Barrett PM, Farrell LJ, Ollendick TH, Dadds M (2006) Long-term outcomes of an Australian universal prevention trial of anxiety and depression symptoms in children and youth: An evaluation of the friends program. Journal of Clinical Child and Adolescent Psychology 35:403–411

91. Boyd CP, Gullone E (1997) An investigation of negative affectivity in Australian adolescents. J Clin Child Psychol 26:190–197

92. Gilmore L, Campbell M, Shochet I (2022) Adapting self-report measures of mental health for children with intellectual disability. J Ment Health Res Intellect Disabil 15:1–19

93. De Ross RL, Gullone E, Chorpita BF (2002) The Revised Child Anxiety and Depression Scale: A psychometric investigation with Australian youth. Behaviour Change 19:90–101

94. Landgraf JM, Abetz L, Ware JE (1996) The Child Health Questionnaire (CHQ): A user’s manual, 1st ed. New England Medical Center, Boston

95. Waters EB, Salmon LA, Wake M, Ch MBB, Wright M, Hesketh KD (2001) The health and well-being of adolescents: A school-based population study of the self-report Child Health Questionnaire. Journal of Adolescent Health 29:140–149

96. Sheehan D V, Lecrubier Y, Sheehan KH, Amorim P, Janavs J, Weiller E, Dunbar GC (1998) The Mini-International Neuropsychiatric Interview (MINI): The development and validation of a structured diagnostic psychiatric interview for DSM-IV and ICD-10. J Clin Psychiatry 59:22–23

97. Sheehan D V, Lecrubier ’ Y, Sheehan H, Janavs J, Weiljer E, Keskiner A, Schinka J, Knapp E, Sheehan MF, Dunbar GC (1997) The validity of the Mini International Neuropsychiatric Interview (MINI) according to the SCID-P and its reliability. European Psychiatry 12:232–241

98. Grant KA, McMahon C, Austin MP (2008) Maternal anxiety during the transition to parenthood: A prospective study. J Affect Disord 108:101–111

99. Law M, Naughton MT, Dhar A, Barton D, Dabscheck E (2014) Validation of two depression screening instruments in a sleep disorders clinic. Journal of Clinical Sleep Medicine 10:683–688

100. Gandy M, Sharpe L, Nicholson Perry K, Miller L, Thayer Z, Boserio J, Mohamed A (2012) Assessing the efficacy of 2 screening measures for depression in people with epilepsy. Neurology 79:371–375

101. Byrne GJ, Pachana NA (2011) Development and validation of a short form of the Geriatric Anxiety Inventory - the GAI-SF. Int Psychogeriatr 23:125–131

102. Tully PJ, Penninx BW (2012) Depression and anxiety among coronary heart disease patients: Can affect dimensions and theory inform diagnostic disorder‐based screening? J Clin Psychol 68:448–461

103. Grech M, Turnbull DA, Wittert GA, Tully PJ (2019) Identifying the internalizing disorder clusters among recently hospitalized cardiovascular disease patients: a receiver operating characteristics study. Front Psychol 10:2829

104. Torsheim T, Wold B (2001) School-related stress, support, and subjective health complaints among early adolescents: A multilevel approach. J Adolesc 24:701–713

105. Sheikh JI, Yesavage JA (1986) Geriatric Depression Scale (GDS): Recent evidence and development of a shorter version. Clinical Gerontologist: The Journal of Aging and Mental Health 5:165–173

106. Davison TE, McCabe MP, Knight T, Mellor D (2012) Biopsychosocial factors related to depression in aged care residents. J Affect Disord 142:290–296

107. Johnson G, Burvill PW, Anderson CS, Jamrozik K, Stewart‐Wynne EG, Chakera TMH (1995) Screening instruments for depression and anxiety following stroke: Experience in the Perth community stroke study. Acta Psychiatr Scand 91:252–257

108. Snowden J (1990) Validity of the Geriatric Depression Scale. J Am Geriatr Soc 38:722–723
